# Nanostructured and bio based high performance adsorbents for the purification of water and food matrices from emerging contaminants

**DOI:** 10.1039/d6ra00667a

**Published:** 2026-07-22

**Authors:** Ghulam Ummul Banin, Suniya Shahzad, Afzal Shah

**Affiliations:** a Department of Chemistry, Quaid-i-Azam University Islamabad 45320 Pakistan afzals_qau@yahoo.com

## Abstract

The prevalent presence of dyes, mycotoxins, and marine toxins in aquatic environments and food products presents significant threats to environmental sustainability and public health. Adsorption has become an effective and adaptable method for pollutant removal owing to its simplicity, high efficiency and adaptability to complicated water and food matrices. This review systematically assesses the efficacy of bio-based and nanostructured adsorbents in the remediation of dyes, mycotoxins, and marine toxins, focusing on adsorption processes, kinetics, and structure–performance correlations. Bio-based materials, comprising biopolymers, agricultural residues, and biohybrid systems are examined in conjunction with nanostructured adsorbents, including carbon nanomaterials, metal oxides and nanocomposites. This review bridges a critical gap in the existing literature by offering a comprehensive cross-disciplinary assessment of aqueous systems and food matrices. It emphasizes how transitions within these matrices influence the efficacy of specific classes of adsorbents across various biological contexts. Ultimately, new trends, existing problems, and prospective research avenues are delineated to facilitate the advancement of sustainable, high-performance adsorbents for applications in water and food safety.

## Abstract 2

The prevalent presence of dyes, mycotoxins, and marine toxins in aquatic environments and food products presents significant threats to environmental sustainability and public health.

## Graphical abstract

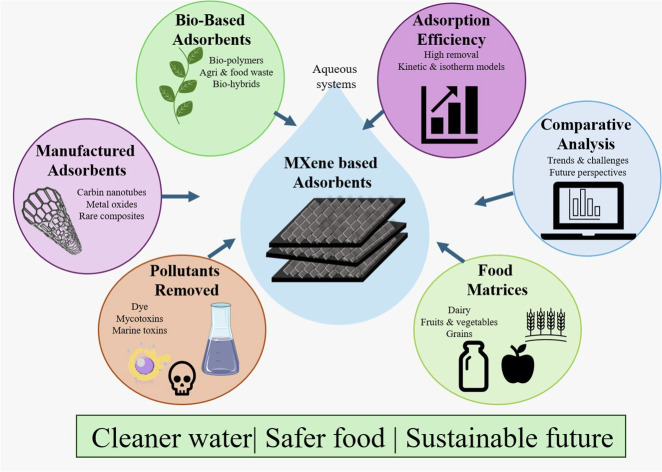

## 1. Introduction

The worldwide challenge of food and water safety primarily stems from the need to control contamination. In the recent decade, the most demanding concern is lack of hygienic and non-toxic food and water as their consumption can lead to a wide range of acute and chronic diseases in both humans and animals.^1,2^ Among the numerous contaminants of concern, mycotoxins, marine biotoxins, and synthetic dyes represent three major classes due to their widespread occurrence, persistence and potential toxicity.^3,4^ Mycotoxins, secondary metabolites produced by filamentous fungi such as *Alternaria*, *Fusarium*, *Aspergillus*, and *Penicillium*, represent a pervasive threat to food safety, since they can contaminate agricultural products and pose serious health risks.^5,6^ An unusual livestock disaster known as “turkey X disease” resulted in death of approximately 100 000 turkey poults, is marked as major scientific turning point to understand threat of mycotoxins. This outbreak led to the identification of aflatoxins a type of mycotoxin, highlighting their lethal potential when the disease was traced to peanut meal contaminated with secondary metabolites produced by *Aspergillus flavus.*^7^ Fungal infections are believed to lead to more than 1.6 million fatalities, with countless others impacted by different fungal diseases. Historical data indicated that approximately 25% of worldwide crops were contaminated, but recent monitoring shows a significantly higher prevalence, with 60–80% of food crops exhibiting measurable levels of mycotoxins.^10^ The economic effect is significant, especially in areas such as India, where aflatoxin contamination leads to agricultural losses that surpass US$932 million each year.^11^ The importance of removing these toxins is underscored by their severe health impacts.

The mycotoxin family is varied and complex, resulting in numerous health hazards. Aflatoxins produced by *Aspergillus flavus* and *A. parasiticus* can contaminate crops such as maize, peanuts, corn, tree nuts, dried fruits, and animal feed and are highly toxic, causing liver cancer, immunosuppression and acute liver toxicity. Ochratoxins are produced by *Aspergillus ochraceus*, *Penicillium verrucous* are nephrotoxic found in cereals (barley, wheat), coffee, wine, pork products, while fumonisins from *Fusarium verticillioides*, *F. Proliferatum* are associated with esophageal cancer, neural tube defects (in animals), pulmonary edema found in corn (maize) and corn-based products. Zearalenone is a toxin produced by the fungus *Fusarium graminearum*, commonly found in corn, wheat, and barley. It can disrupt hormones (endocrine system) and cause reproductive problems in both humans and animals. Another toxin, namely patulin, produced by the fungus *Penicillium expansum* is usually present in moldy apples, and can cause gastrointestinal problems such as nausea and ulcers. It is also considered a potential carcinogen.^8^ Because of their hazardous nature, the International Agency for Research on Cancer categorized them as Group 1 human carcinogens.^9^ For public safety, the European Union has established maximum permissible limits as specified in Regulation No. 915/2023. These standards consist of 2.0 µg kg^−1^ for aflatoxin B1 in cereals and 0.050 µg kg^−1^ for aflatoxin M_1_ in raw milk, which is subsequently lowered to 0.025 µg kg^−1^ in infant formula. Moreover, ochratoxin A is controlled at 10.0 µg kg^−1^ in dried grape products and 0.50 µg kg^−1^ in infant cereals.^10^

Mycotoxins affect sexual health, particularly through the contamination of food sources that pose risks to vulnerable populations. Clinical evidence reveals presence of high levels of Ochratoxin A (OTA) in 70% of infertile men and aflatoxin in 60% of men, and a leading cause of miscarriage in expecting females.^11^ These reproductive and systemic effects are driven by complex molecular processes such as inhibition of protein synthesis, lipid peroxidation and mitochondrial impairment. These disruptions collectively trigger oxidative stress and lead to irreversible DNA damage. In severe cases, like the major aflatoxicosis outbreak in Kenya, contact with mycotoxins has resulted in more than 150 deaths in just one year.^12,13^ The presence of major mycotoxins, such as aflatoxins (AFs), ochratoxin A (OTA), patulin (PAT), deoxynivalenol (DON), fumonisins (FBs), and zearalenone (ZEN) in processed infant foods represents a critical global concern with serious health implications for children.^14–16^ Unfortunately, it is impossible to eradicate the formation of mycotoxins in food due to the complex storage environments, poor harvesting practices, inappropriate storage practices by consumers and improper transport conditions, resulting in a pervasive problem in the food industry. Therefore, decontamination of mycotoxins is a challenging issue and various strategies based on physics, chemistry and biology have been developed for effective detoxification of mycotoxins. Among these, adsorption method seems to be a promising direction to achieve the reduction in mycotoxins owing to its simple operation, high removal efficiency and low cost.^17^

Marine biotoxins, much like mycotoxins, pose a significant threat that requires effective intervention strategies. These toxins can accumulate in the food chain, resulting in severe health risks for humans. While seafood is celebrated for its health benefits, including vital minerals such as calcium, phosphorus, and iron, it is also a primary source of omega-3 fatty acids like eicosatetraenoic acid and docosahexaenoic acid (DHA), essential for cell membrane integrity. However, the consumption of seafood carries inherent risks, including food poisoning from allergic reactions or the presence of marine toxins.^18^ These biotoxins, produced by phytoplankton, can be classified into hydrophilic and lipophilic compounds. Hydrophilic toxins include domoic acid, saxitoxins, and tetrodotoxins, while lipophilic toxins comprise okadaic acid, dinophysis toxins, azaspiracids, yessotoxins, and spirolides. Although most marine toxins are cell-bound, some are water-soluble.^19^ Biotoxins generated from algal blooms are responsible for an estimated 60 000 human poisoning cases worldwide every year, along with many other fatalities. In case of ciguatera poisoning, available epidemiological information indicates that the officially noted range of 10 000 to 50 000 cases each year likely reflects fewer than 20% of the real incidents, mainly because of considerable underreporting, particularly in regions where the disease is not widespread.^20,21^ The ingestion of contaminated seafood can lead to various toxic effects in humans, including paralytic, neurotoxic, amnesic, and diarrheic syndromes. Consequently, there is an urgent need for more effective and adaptable intervention strategies.

Marine biotoxins are a diverse group of natural metabolites produced by various phytoplankton, including dinoflagellates like *Alexandrium* spp. and *Karenia brevis*, diatoms such as *Pseudo-nitzschia* spp., and cyanobacteria. These toxins accumulate in filter-feeding organisms, particularly shellfish and pelagic fish, ultimately entering the human food chain and leading to serious health issues, including amnesic shellfish (ASP), diarrhetic shellfish poisoning (DSP), paralytic shellfish poisoning (PSP), neurotoxic shellfish poisoning (NSP), azaspiracid poisoning (AZP), ciguatera fish poisoning (CFP), as well as microcystins and tetrodotoxin poisoning. Historical incidents of mass poisoning, such as the 1987 ASP outbreak in Canada linked to domoic acid and recurrent cases of CFP in regions like French Polynesia and Hawaii, highlight significant weaknesses in global food safety and monitoring systems, leading to the implementation of some of the world's strictest food safety regulations.^22^ Regulatory authorities have established strict maximum levels for algal toxins in shellfish. The allowed limits include 20 mg eq. kg^−1^ for domoic acid, 0.8 mg eq. kg^−1^ for saxitoxins and 0.16 mg eq. kg^−1^ for okadaic acid. Moreover, yessotoxins are regulated in EU at 3.75 mg eq. kg^−1^, while Brazil enforces a more stringent limit of 1.0 mg eq. kg^−1^ in certain regions.^20^ In addition to the threat posed by mycotoxins^11^ and marine biotoxins, synthetic dyes are also harmful. Their widespread use in various industries results in their release into the environment alongside other inorganic and organic pollutants.

Numerous physical, chemical, and biological treatments are required to safeguard the environment from dye effluents produced in water. The water bodies are polluted by the discharge of contaminated water by consuming or producing units of dyes and constitute a possible threat particularly to human health, plants and animals, and in general to aquatic biota. Even in minute concentrations, the presence of colors in wastewater is very unpleasant and unwanted. Azo dyes, often present in food items, have aromatic centers within their molecular composition. The metabolic and breakdown products of these dyes, including aromatic amines, benzene sulfonic acids, and benzidines, are recognized as mutagenic and carcinogenic, resulting in cellular damage. Furthermore, dyes can color water, limiting transparency (sunlight penetration) and aeration of water bodies, thus impacting the effectiveness of critical photosynthesis and, as a result, drastically lowering dissolved oxygen (DO) levels in the water. Dye effluents discharged into bodies of water have both direct and indirect effects on aquatic ecosystems.^23,24^ Due to the increased demand for coloring and fluorescence agents, synthetic dyes such as RhB have almost completely replaced natural dyes, particularly in fabric, paper making, textile, and many other industries. In the long term, industrial production and discharge of wastewater containing dyes have seriously threatened living organisms' survival and disrupted the ecological through genetic disruptions. They are known to cause skin irritations, thyroid, and liver damage. These dyes are carcinogenic, mutagenic, non-degradable and are thus known to cause cancer to humans.^25,26^ Various physical, chemical and biological processes have been employed for dyes, mycotoxins and marine toxins removal from wastewater.

Several technologies, such as adsorption, coagulation, advanced oxidation, membrane separation, biological treatments, and ozonation, can be used to remove dyes, marine toxins and mycotoxins. Out of all these, adsorption is highlighted in this review for its advantages over other conventional methods. Adsorptions are one of the most effective advanced wastewater treatments for the removal of hazardous inorganic and organic pollutants from effluents due to its ease of operation, high efficiency, and low cost. At its core, adsorption is a process where attractive forces associate a solute (adsorbate) to the surface of an adsorbent until equilibrium in the concentration of the adsorbate is attained and no further net adsorption occurs. It could also be defined as the formation of layer of adsorbate on the surface of an adsorbent by the attraction of van der Waals' forces. The process involves the accumulation of adsorbate at an interface relative to its concentration in the bulk solution. Adsorption could be physical (physisorption) or chemical (chemisorption). Physical adsorption is a result of weak, short range electrostatic. Attractive forces arising from dipole moment (van der Waals); and chemisorption involves bond formation between the adsorbate and the adsorbent.^27^ The term bio-sorption is used for the type of adsorption in which the adsorbate adsorbs on natural materials (biological systems).^28^ The effectiveness of this process is governed by key factors like pH, temperature, contact time, porosity of adsorbent, adsorbent dose and initial contaminant concentration. In fact, the particle size of adsorbent and synthesis route by which adsorbent is prepared also influenced the adsorption process.^29^ These interactions are mathematically described by kinetic and isotherm models, which are crucial for understanding the mechanism and efficiency of any adsorbent.

This review specifically focuses on nanostructured and bio-based adsorbents because they represent the frontier of adsorption technology, moving beyond traditional materials like activated carbon. An ideal adsorbent should be nontoxic, environmentally safe and should not form permanent bond with contaminant molecules.^12^ Additionally, it should be easy to regenerate and have strong selectivity and sensitivity, even when contaminants are in low concentrations. In the last decades, nanotechnology has become a promising tool for the treatment of water and food contaminated with different compounds. Nanostructured adsorbents are chosen for their exceptional surface area, with graphene derivatives attaining theoretical values as high as 2630 m^2^ g^−1^, and for their tunable surface chemistry, which improves adsorption capacity. These carbon-derived materials are characterized by sp^2^ hybridized atoms and elevated carrier mobility, facilitating efficient π–π interactions with aromatic pollutants, which renders them especially appropriate for handling organic loads.^30^

Bio-based adsorbents are selected for their sustainability, low cost, and biodegradability, aligning with green chemistry principles. The field is currently shifting toward sustainable carbon materials that prioritize low-energy synthesis for the removal of heavy metals and radionuclides.^31^ Furthermore, a waste to resource paradigm has emerged describing that upcycling plastic waste into functional nanomaterials of various dimensions including 0 D quantum dots, 1D nanotube and 3D porous frameworks provides high-performance solutions for capturing antibiotics and atmospheric CO_2_.^32^ To prioritize these complex systems, artificial intelligence is now integrated to predict structure–property relationships and accelerate the discovery of matrix-resilient adsorbents.^31^ This review presents a novel perspective by emphasizing that emerging contaminants extend beyond just food or environmental systems. Rather, these contaminants display considerable mobility and can persist in both food and water. This observation supports the integrated “one health perspective,” highlighting the necessity for a comprehensive strategy to comprehend and address these pollutants. Unlike previous reviews that address industrial wastewater^1,33^ and food safety^17,34^ as separate issues, this study adopts an integrated, cross-matrix approach. This perspective is scientifically significant as it helps in identifying chemical functionalities that remain effective across different matrices. By analyzing both simple aqueous media and complex biological matrices rich in lipids and proteins,^35^ this study points out which nanostructured materials can retain high selectivity even under conditions of strong competition and surface fouling. Based on these insights, this review introduces a ternary hybridization framework that integrates a biological foundation, nano-functionalization and magnetic recovery. This framework strategically aims to guide researchers in designing advanced adsorbents capable of effectively removing a wide range of contaminants across diverse systems.^36^

Recent review articles have examined adsorbent materials for contaminant removal, often concentrating on particular categories such as polymer-based systems, nanomaterials, or wastewater treatment applications. For example, several studies have reported progress in the development of polymeric adsorbents, nanostructured materials, and hybrid systems for environmental remediation.^37^ Nevertheless, these analyses tend to remain confined either to water treatment contexts or to narrowly defined material classes, without considering the combined performance of nanostructured and bio-based adsorbents in both aqueous environments and more complex food matrices. By comparison, the present review adopts a more comprehensive approach, assessing adsorption efficiency alongside mechanistic understanding and practical considerations including regeneration potential, scalability, and real-world limitations, thereby presenting a broader and more integrated perspective on emerging contaminants.^38,39^

Therefore, the scope of this work is to consolidate and evaluate the existing knowledge on these advanced materials. In this regard, several nano structured adsorbents have been proposed for the detoxification of contaminated water with a huge variety of compounds such as mycotoxins, marine toxins and dyes. There is a wide variety of adsorbents applied for the removal of dyes from wastewater including clays minerals, zeolites, composites, various agro-industrial wastes, inorganic nanomaterials, metal–organic frameworks and metal oxides. Various synthetic strategies and potential of nanomaterials, particularly carbon-based and metal oxide-based nano-adsorbents are highlighted, for effective treatment of dye-contaminated wastewater, offering advanced and cost-effective solutions for water purification. Additionally, this article provides a comprehensive discussion on a multitude of aspects encompassing the adsorption kinetics and isotherms, while emphasizing the different operating parameters associated with the adsorption process and the renderability of the fabricated adsorbents. Dedicated sections focused on the real-world applications, environmental fate and toxicity, and economic feasibility of these nanomaterials have been extensively presented. Finally, the challenges associated with the practical implementation of these nano-adsorbents, as revealed in the literature, have been addressed, proposing ways for future advancements for addressing these challenges.

### 1.1. Adsorption mechanism and kinetic models

**Fig. 1:**
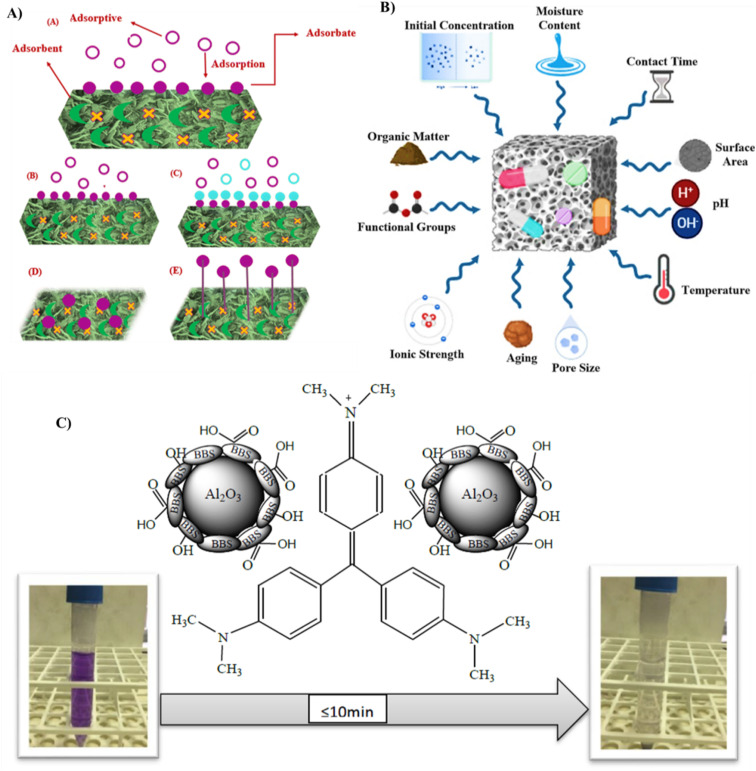
(A) Schematic representation of the sequential steps involved in the adsorption of environmental contaminants, including bulk diffusion, external mass transfer to the adsorbent surface, intraparticle diffusion within pores, and surface interaction with active sites, highlighting the key physicochemical processes governing adsorption efficiency. Reproduced with permission from ref. 29 (B) schematic illustration of the key physicochemical factors influencing adsorption performance, including initial contaminant concentration, pH, temperature, contact time, ionic strength, moisture content, organic matter, surface area, pore size, aging effects, and surface functional groups, highlighting their collective impact on adsorption capacity, kinetics, and interaction mechanisms. Reproduced with permission from ref. 40 and, (C) schematic representation of the adsorption mechanism of dye molecules onto functionalized Al_2_O_3_-based adsorbents, illustrating surface complexation and electrostatic interactions between dye molecules and hydroxyl-functionalized sites, accompanied by photographic evidence demonstrating rapid decolorization of the dye solution within ≤10 min. Reproduced with permission from ref. 41.

Adsorption technique is one of the most effective options due to the cost-effectiveness, ease of operation, high removal efficiency and regeneration of adsorbents. Adsorption process generally involves these four stages, (1) diffusion of molecules from the fluid to the fluid boundary layer near the adsorbent, (2) diffusion through the fluid boundary layer, (3) adsorption onto the active sites, and finally, (4) intraparticle diffusion within pores. Adsorption is generally divided into two main types, physisorption and chemisorption. Fig. 1A illustrates the general mechanism of adsorption (Fig. 1A(A)), with key steps including monolayer adsorption (Fig. 1A(B)), multilayer adsorption (Fig. 1A(C)), physical adsorption (Fig. 1A(D)), and chemical adsorption (Fig. 1A(E)). In physisorption, the adsorbate attaches to the adsorbent surface through weak van der Waals interactions, making the process reversible and non-specific in nature. In contrast, chemisorption involves the formation of stronger ionic or covalent bonds between the adsorbate and adsorbent, leading to irreversible adsorption due to chemical reactions. Adsorption can occur on both synthetic and natural materials, while the term biosorption specifically refers to adsorption processes in which natural or biological materials act as the adsorbents.^15^ Various factors intricately affect the adsorption process of adsorbates onto nanosorbents. These encompass the pH level of the solution and the dimensions of the pores of the nanoadsorbent, potential ionic interference from the environment, the chemical composition of the pharmaceutical compound, the reactive functionalities on the adsorbent's surface, the temperature during the process, the contact duration, the agitation intensity, the specific surface area of the adsorbent, and the initial concentration of the adsorbate substance, among others (Fig. 1B).^16^ In a study γ-Al_2_O_3_-bio-based substance (A-BBS) hybrids were prepared *via* simple electrostatic interactions between alumina and compost-derived BBS (bio-based substance) (Fig. 1C). The optimal BBS loading and hybrid stability were determined to enable effective adsorption of polar pollutants. Comprehensive characterization confirmed successful BBS immobilization and favorable surface properties. Adsorption studies using crystal violet and emerging contaminants demonstrated that A-BBS is a promising, environmentally sustainable adsorbent for wastewater treatment.^17^

During the removal of pollutants using adsorbents, adsorption generally occurs by either physical adsorption or chemical adsorption. Various interaction methods may contribute to uptake contaminants from contaminated matrix including π–π interactions, hydrogen bonding, van der Waals forces, electrostatic attraction, hydrophobic interactions, and acid–base interactions. For instance, the adsorption of Rhodamine dye on the surface of the adsorbent is largely driven by electrostatic and intermolecular interactions. In ion exchange adsorption, the ions from contaminant solution are exchanged with ions present on adsorbent surface. So, this process relies on binding of ions to specific surface bonding groups in addition to electrostatic forces between contaminant and the adsorbent surface. Hydrogen bonding interactions have also been reported in the adsorption of acid orange 7 dye on various adsorbents. Likewise, π–π interactions involve interaction between positively charged contaminant molecules and negatively charged adsorbent surfaces, like non-covalent interactions. In multiple cases, many adsorption mechanisms can operate simultaneously. A critical mechanistic comparison shows that different classes of adsorbents rely on distinct interaction pathways. As demonstrated in various studies, traditional activated carbon primarily operates through pore filling and hydrophobic interactions,^42,43^ while bio-based materials depend largely on ion exchange *via* hydroxyl and amino groups and surface complexation.^44,45^ In contrast, carbon based nanostructured materials such as carbon nanotubes and graphene utilize delocalized π-electrons for strong π–π interactions with aromatic contaminants.^21,46^ So adsorbent design must be mechanistically matched to the target contaminant. Based on these interaction principles, recent research on KOH-modified silica sand elaborated that the removal of amoxicillin follows the Langmuir model, which shows that chemical modification can successfully create a uniform monolayer for pharmaceutical capture.^31^ Although electrostatic interactions may be sufficient for small ionic dyes,^33^ the effective removal of larger and multifunctional pollutants requires combined mechanism including hydrogen bonding and multipoint surface complexation.^17,47^

Integrating adsorption mechanism across these three pollutant classes reveals both common and distinct interaction pathways. The aromatic nature of mycotoxins, dyes and marine toxins leads π–π interactions to function as a fundamental adsorption mechanism specifically on carbon-based adsorbents. However, dissimilarities in molecular structure govern the prevalence of secondary interactions. Synthetic dyes typically ionic and small sized are primarily adsorbed through pore filling and electrostatic interaction.^33^ Conversely mycotoxins such as AFB1 contain rich oxygen functionalities that support strong multipoint hydrogen bonding along with van der Waals interactions.^48^ Marine toxins display the greatest structural variability, smaller, polar species are inclined to undergo ion–exchange interactions, while larger, lipophilic phycotoxins rely on hydrophobic partitioning into non-polar domains.^22^ These insights highlight that a ternary hybridization strategy is not merely advantageous but significant as it integrates aromatic, ionic and hydrophilic functionalities to effectively capture structurally diverse contaminants within a single adsorptive system.

**Fig. 2:**
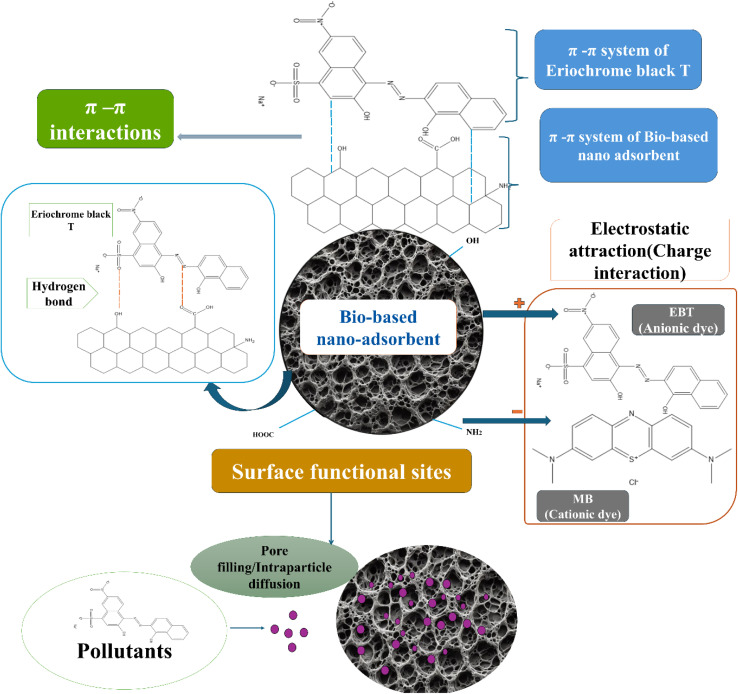
Schematic representation of the multiple adsorption mechanisms involved in the removal of emerging contaminants by bio-based and nanostructured adsorbents. The mechanistic interactions include electrostatic, hydrogen bonding, π–π interactions, and pore-filling/diffusion processes, highlighting the role of surface functional groups and structural properties for enhanced adsorption performance.

Adsorption typically occurs when the pollutant exhibits low solubility in the surrounding medium or when it shows a stronger affinity for the adsorbent surface. Adsorption occurs because the surface particles of the adsorbent hold unbalanced attractive forces that are not in the same condition as the particles in the bulk adsorbent, which have all the forces balanced together. The energy of the particles near the adsorbent's surface is substantially higher than that of the particles in the bulk. Surface energy is the extra energy per unit of surface area that is required for the attraction of adsorbate to its adsorbent surface. At a given setting, the degree of adsorption tends to increase as the surface area of the adsorbent per unit mass increases.^23^ As illustrated in Fig. 2, adsorption of emerging contaminants is governed by multiple simultaneous mechanisms, including electrostatic interactions, hydrogen bonding, π–π stacking, and intraparticle diffusion, depending on the physicochemical properties of both the adsorbent and the target pollutant. Overall, adsorption is recognized as a versatile, sustainable, scalable and practical method for addressing environmental pollutants, marine toxins and mycotoxins.

Isotherm studies explore the homogeneity and heterogeneity of adsorbents. An adsorption isotherm represents the relationship between the amount of pollutant adsorbed and its concentration in water at equilibrium. Literature has presented several models of adsorption isotherms, such as Henry, Freundlich, Langmuir, Brunauer–Emmett–Teller (BET), Temkin, Frumkin, Redlich–Peterson, Radke–Prausnitz, and Dubinin–Radushkevich (D–R). The Freundlich model describes an empirical equation assuming an exponential energy distribution of the adsorption sites, which is applied to the description of multilayer adsorption on the heterogeneous surface. The Freundlich model only provides a good representation of the experimental data in the moderate concentration range. This model is represented in eqn (1), where *C*_e_ (mg L^−1^) is the equilibrium concentration of the solute *K*_f_ ((mg g^−1^) (L mg^−1^)1/*n*) and *n* (dimensionless) are the constants.^34^

1 *q*_e_ = *K*_f_*C*_e_^1/*n*^

In the Langmuir model, the adsorption process is based on a reversible chemical reaction where the adsorption and desorption rates are equal at equilibrium. This model is used to quantify the adsorption performance of gases on a solid surface. In these cases, monolayer adsorption occurs on a structurally homogeneous surface; each adsorption site can hold only one adsorbate species; all adsorption sites are energetically equivalent and independent; and there is no interaction between adsorbate molecules adsorbed on neighboring sites. This model is represented in eqn (2), where, *C*_e_ (mg L^−1^) is the equilibrium concentration of the solute, *q*_max_ is the theoretical maximum monolayer adsorption capacity (mg g^−1^) obtained through model fitting whereas *q*_e_ represents experimental adsorption capacity at equilibrium and *K*_L_ is the Langmuir constant (L mg^−1^).2
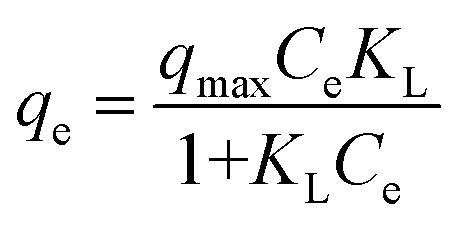


Eqn (3) relates how much of the surface is covered (*θ*) to the equilibrium concentration, accounting for different adsorption energies or site heterogeneity. In the Temkin isotherm, *q*_e_ (mg g^−1^) represents the equilibrium adsorption capacity, *q*_max_ is the maximum adsorption capacity, *C*_e_ (mg L^−1^) the equilibrium concentration of the adsorbate, *R* the universal gas constant (8.314 J mol^−1^ K^−1^), *T* the absolute temperature (K), *b* the Temkin constant related to the heat of adsorption (J mol^−1^), and *K*_*T*_ the equilibrium binding constant (L g^−1^).^49,50^3
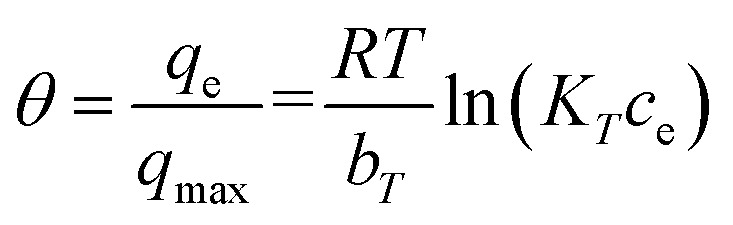


The Langmuir model is often inadequate for describing equilibrium data in aqueous solutions, as its underlying assumptions, such as monolayer coverage of the adsorbent surface and energetic homogeneity of adsorption sites, are not met. Additionally, the Freundlich model is applicable only within a moderate concentration range. The three-parameter empirical model combines aspects of the Langmuir and Freundlich models. The Redlich–Peterson model follows Henry's law at low concentrations and approximates the Freundlich model at high concentrations.^34^ Thus, it can be used to describe adsorption behaviors in low to moderate concentration ranges described in eqn (4), where *K*_RP_ (L g^−1^) and *a*_RP_ (L mg^−1^) are Redlich–Peterson constants and *β* exponent in the range of 0 and 1 reflects system heterogeneity.4
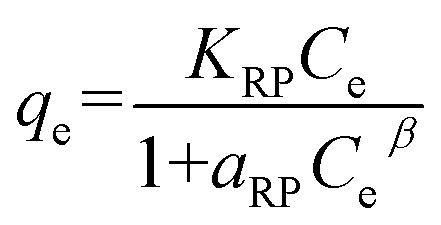


The Dubinin–Radushkevich (D–R) model is commonly used to determine whether adsorption is physical or chemical. The non-linear equation is given as

5 *q*_e_ = *q*_max_*e*^{−*β* [*RT*ln(*C*_s_/*C*_e_)]^2^}^

The symbol “*C*_s_” represents the saturation concentration of adsorbate. To ensure the validity of model, *C*_s_ must be used as fixed constant. In this equation, *β* is the activity coefficient having units as mol^2^ J^−2^, *R* is general gas constant expressed in J K^−1^ mol^−1^ and *T* is the absolute temperature in kelvin. To maintain physical consistency and accuracy in model parameters, this standard form is preferred over the empirical one for D–R model. However, a critical analysis of the literature reveals that many documented studies have reported D–R model in a simplified but flawed form where *C*_s_ is arbitrarily assumed to be 1.^51^ This practice introduces a significant methodological error that leads to dimensional inconsistencies. Using a unit dependent 1 instead of the actual physical solubility makes other parameters such as adsorption potential (*ε*) and the activity coefficient (*β*) mathematically invalid across different concentration units. Consequently, this leads to errors in the calculations of further derived parameters; therefore, conclusions in literature based on uncorrected saturation concentration (*C*_s_) values must be interpreted with caution to maintain scientific integrity. Actual *C*_s_ values of adsorbate must be used to avoid errors.^49^

It is essential to address a frequent misconception in adsorption studies regarding the interpretation of kinetic models. Although a good fit to PSO model is often interpreted as evidence of chemisorption, but this review highlights there is no theoretical relationship between PSO model and nature of adsorption bond. The PSO is an empirical mathematical equation that provides the description of adsorption rate and cannot be used to infer the underlying mechanism.

The linear representations of kinetic and isothermal models are often used for analysis; however, they are inadequate in delivering accurate estimates of the parameters. Conversely, nonlinear regression provides a method for estimating model parameters that are not only scientifically sound but also yield greater accuracy. This is essential as linearizing equations can change the initial error distribution, which may introduce biases in the computed adsorption capacity and rate constants. While non-linear regression is often cited for its mathematical accuracy in parameter estimation, linear transformations remain a widely accepted practice among renowned authors in the field of adsorption. In this review we report data from both methodologies to ensure completeness. To address mathematical inconsistencies, we report *R*^2^ values only for linear models as provided in the original literature while relying strictly on the absolute adsorption capacities values for the comparison among all adsorbents. This ensures that the evaluation is based on physically meaningful parameters rather than incomparable correlation coefficients.

## 2. Biobased adsorbents

The global research community is increasingly focused on the advancement of natural biobased materials aimed at preserving endangered ecosystems. Microorganism-based biological adsorption has shown effectiveness in the extraction of mycotoxins, marine toxins and dyes. Furthermore, bio-based materials are recognized as ideal for water purification due to the presence of critical functional groups like carboxyl, hydroxyl and amino groups. However, it is important to note that single-source biomass often faces limitations such as chemical instability and weak chemical strength which necessitates chemical modifications to enhance its industrial performance.^52^ Yeast cell wall components, particularly β-glucan and mannan oligosaccharides, have been identified to increase binding capacity for mycotoxins.^53^ Biosorption is widely acknowledged as an effective treatment due to its low cost, operational simplicity and eco-friendly nature. Some of the common biomaterials are natural polymers, bacteria and algae, which are typically sourced from industrial and agricultural waste. These biomaterials possess a diverse array of functional groups that can effectively bind both ionic and non-ionic pollutants, underscoring the practical application of such biosorbent materials.^44^

### 2.1. Biopolymers

Renewable biopolymers (chitosan, keratin, and cellulose) are viable and biodegradable adsorbents. Chitosan (CS), which is an extracellular degradable biopolymer (derived from chitin), has shown potential in adsorption of mycotoxins due to its combination of properties, such as solubility in water, biodegradability, and adsorption capacity, lack of toxicity, antimicrobial activity, solubility in water, bioactivity, and chemical reactivity. The CS matrix is a carbon-based framework containing hydrogen atoms and has hydroxyl groups and amino groups as active sites, which permit electrostatic interactions with anionic dyes. CS is, however, not a perfect adsorbent due to its pH dependent aqueous solubility, gelation in acidic solutions, limited mechanical and thermal activity, and surface area size.^44^ A recent study proves the usefulness of CS and its derivatives, especially nano CS, in the adsorption and detoxification of aflatoxins. The research indicates that both CS and nano CS exhibit significant adsorption capabilities, achieving a peak adsorption rate of 74.77 percent when combined with metal oxide-based materials (MOS) and Bentonite (Bn) in a 1 : 1 : 1 ratio. The adsorption efficiency is influenced by pH levels, with slightly acidic conditions yielding the best results. Furthermore, the use of CS as an adsorbent is economically advantageous, positioning it as a viable solution for the feed and food industries.^54^

Another study developed a fast, green and efficient adsorbent using lignin modified or grafted with CS and iron nanoparticles (Fe-NPs). This approach resulted in the significant increase of aflatoxin B_1_ (AFB_1_) removal capacity in a batch reactor setting. Notably all materials used in the synthesis process were sourced in agro-waste, making this method not only cost effective and reliable but also environmentally friendly and sustainable. A critical analysis of the AFB_1_ adsorption revealed that the adsorption to the surface of the adsorbent is dependent on the dose/concentration of AFB_1_, pH, time and temperature. The ultimate nanocomposite of L-CS-Fe depicted the highest adsorption of AFB_1_ up to 95.6% using a small quantity of adsorbent (1 mg mL^−1^) and a broad spectrum of structural stability. The adsorptive capacity was improved through synergistic effects caused by the composite formation of lignin, chitosan and iron, and capitalized on the individual strengths of each constituent, lignin adsorption capacity, chitosan biodegradability and chelation properties and iron reactive capability. Notably, aminated lignin produced *via* the Mannich reaction has achieved over 90% removal efficiency for phosphates. Additionally, starch-based hybrids are proving to be increasingly effective in the selective adsorption of complex azo dyes, such as Ponceau Red, which traditional biopolymers often fail to capture effectively.^52^ Equilibrium modeling indicates that the adsorption process follows Langmuir isotherm model, suggesting monolayer adsorption.^55^ While complex composites show great promise, researchers are also exploring simpler, naturally occurring biopolymers for mycotoxin removal. The results of an experiment show that the ability of β-d-glucan produced by pleurotus ostreatus to absorb AFM_1_ in the milk is better than other methods. It effectively removed AFM1 at low levels (35 ng L^−1^) and significantly reduced AFM1 (by about 70%) to higher levels (120 ng L^−1^) in contaminated milk. In addition, β-d-glucan from *P. ostreatus* is easy to obtain because the fungal mycelium can be grown in bioreactors or produced using food-industry waste materials. While the use of β-d-glucan derived from pleurotus ostreatus to adsorb AFM1, may alter the sensory properties of milk such as color, odor or taste of the milk, yet this material also provides beneficial bioactive properties.^56^

Biomaterials sourced from biopolymers for dye elimination are ecologically sustainable across their complete life cycle, including raw material extraction, manufacturing, and usage. These materials provide unique advantages, such as improved characteristics concerning surface area, pore size, and pore volume, along with convenience in handling. Furthermore, they reflect a strong commitment to environmental stewardship, making them a responsible choice for addressing dye pollution. The continuous discharge of toxic dyes such as crystal violet (CV) into aquatic systems represents a serious environmental concern due to their persistence, toxicity, and resistance to biodegradation. Recently, a bio-catalytic strategy based on horseradish peroxidase (HRP) immobilized on a cationic cellulose aerogel (CATCE-HRP) has been reported as an efficient and sustainable platform for CV degradation under visible-light irradiation. The cationic cellulose aerogel provides a highly porous, biocompatible matrix that enhances enzyme stability, facilitates electrostatic interactions with cationic dye molecules, and improves catalytic efficiency. Compared to free HRP, the immobilized enzyme exhibited markedly enhanced thermal and pH stability, prolonged storage life, and excellent reusability. Under optimized conditions, complete CV degradation was achieved within a short reaction time, following pseudo-first-order kinetics. Statistical optimization further demonstrated effective dye removal under mild operational conditions. Importantly, toxicity assessment using the Microtox® bioassay confirmed a substantial reduction in effluent toxicity after treatment, indicating successful detoxification.

**Fig. 3:**
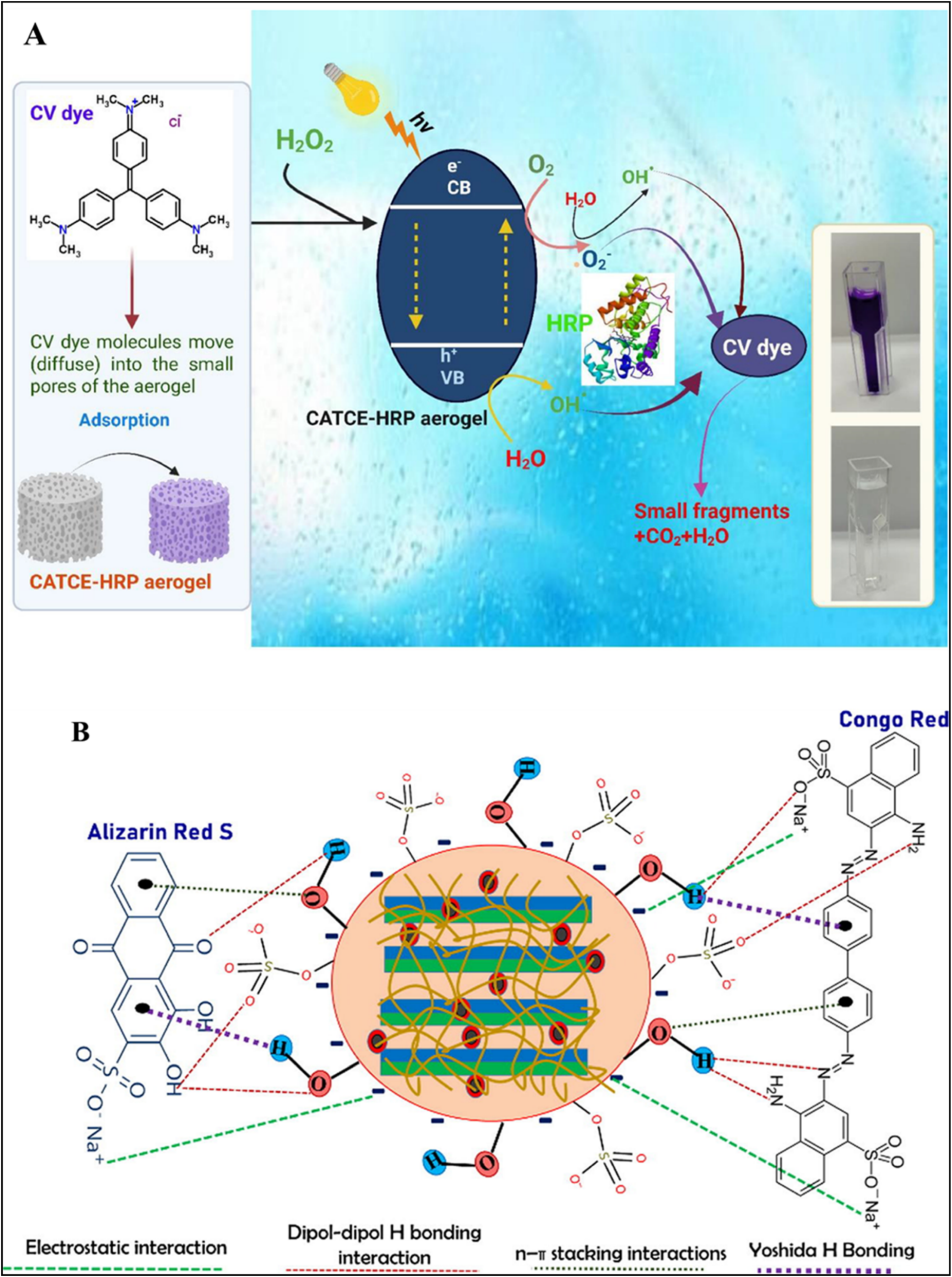
(A) Proposed reaction mechanism for the visible-light-driven photocatalytic degradation of CV dye using CATCE-HRP aerogel (0.5 g L^−1^), illustrating the irradiation driven generation of reactive oxygen species (˙OH, O_2_˙^−^) and their role in dye mineralization. Reproduced with permission from ref. 57 (B) schematic representation of the adsorption mechanisms of CR and alizarin red S onto CKAlFe, highlighting electrostatic interactions, hydrogen bonding and surface complexation between dye molecules and active functional sites on the adsorbent. Reproduced with permission from ref. 58.

Mechanistically, (illustrated in Fig. 3A) the photocatalytic behaviour of the CATCE-HRP aerogel differs fundamentally from that of conventional semiconductor systems. Instead of electron–hole pair generation, CV degradation proceeds through HRP-mediated oxidation in the presence of H_2_O_2(aq)_, with visible light promoting electron transfer between the enzyme and substrate and enhancing the formation of reactive oxidative species such as ˙OH radicals. These intermediates attack the conjugated chromophoric structure of CV, leading to aromatic ring cleavage, decolorization, and progressive mineralization into less toxic products. Overall, this work highlights enzyme-based aerogel systems as promising eco-friendly and scalable alternatives for advanced dye wastewater treatment.^57^

The fabrication of the CKAlFe adsorbent was carried out by synthesizing magnetic Fe_3_O_4_ nanoparticles, which are subsequently encapsulated within an Al_2_O_3_ layer formed *via* the hydrolytic decomposition of aluminum isopropoxide in a mixed water–ethanol medium. The stability of this core–shell architecture is mainly ensured by weak intermolecular (van der Waals) interactions, which also contribute active surface sites for adsorption. The resulting coated nanoparticles are then combined with kaolinite clay and the biopolymer *κ*-carrageenan to construct the composite hydrogel. In this assembly stage, the polymer chains infiltrate the two-dimensional lamellar structure of kaolinite, causing a partial widening of the interlayer spacing through an intercalation process. The final material was stabilized by a cooperative network of hydrogen bonding and van der Waals interactions, in which *κ*-carrageenan forms the continuous amorphous phase, while kaolinite, alumina, and magnetite act as the crystalline components within a plate-like, microporous hydrogel framework.^58^ As illustrated in Fig. 3B, the magnetic hydrogel functions as an efficient platform for the concurrent uptake of Congo Red (CR) and Alizarin Red S (ARS), where adsorption is driven by a combination of complementary intermolecular forces. A key contribution arises from electrostatic attraction between the negatively charged functional groups of the anionic dyes and oppositely charged sites on the adsorbent surface. In addition, strong π–π interactions occur between the aromatic ring systems of the dye molecules and the conjugated domains within the hydrogel structure. Hydrogen-bonding interactions further enhance dye immobilization, involving dipole–dipole associations between dye functional moieties—such as sulfonate (–SO_3_^−^), hydroxyl (–OH), azo (–N=N–), and amine (–NH_2_) groups—and the hydroxyl or sulfate functionalities present on the CKAlFe surface. Moreover, Yoshida-type hydrogen bonding plays a significant role, enabling specific interactions between the aromatic rings of the dyes and hydrogen atoms of surface hydroxyl groups.^25^

Synthetic scheme for the preparation of CGGO hydrogel particles for HM adsorption was described by Perumal, Suguna, *et al.* in which chitosan/gelatin (CG) hydrogel particles were prepared using different chitosan-to-gelatin ratios. Chitosan (3 wt% in 0.1 M acetic acid), gelatin (10 wt% in water), and GO (0.1 wt% aqueous dispersion) solutions were prepared separately and then added to a Span 85/hexane solution at 45 °C, followed by stirring at 550 rpm for 2 h. GO dispersion was sonicated for 1 h prior to mixing. After 2 h of stirring, glutaraldehyde was added dropwise, and the mixture was stirred for an additional 30 min to complete crosslinking. These hydrogels proved to be efficient for adsorption of heavy metal ions such as Pb(ii), Cd(ii), Hg(ii), and Cr(iii).^59^ ZnO in the form of flower was synthesized using biopolymers cellulose and chitosan through biomimetic strategy in a mild alkaline solution, and it was observed that the bandgap of the semiconductor was tuned, which caused the additional enhancement of photocatalytic properties. 87% of methyl orange (MO) was degraded in cellulose-chitosan-ZnO or CCZ nanocomposite in 50 min.^60^ The fabrication of these advanced biopolymer-based adsorbents often involves self-assembly or chemical crosslinking to create robust structures like hydrogels.

Another study used a biopolymer sponge (PS) as a crosslinked gelatin-chitosan-poly (vinyl alcohol) mixture and dipped the sponge into graphene oxide (GO) solution to create a composite sponge (CS, GO-coated PS) through a series of simple dipping and freeze-drying steps, as a sponge-like adsorbent in the removal of organic dyes *i.e.* Rhodamine B and Congo red. The highest adsorption capacity of the Rhodamine B and Congo red was 126.8 and 135.0 mg g^−1^; 145.6 mg g^−1^ and 148.6 mg g^−1^ on the PS and CS respectively. It is noteworthy that the adsorbents in the form of sponges could be reused after being regenerated without an impressive decline in the dye removal capacity after 9 adsorbing–desorbing cycles.^61^ Moving from sponge-like structures to hydrogels, another study prepared efficient biosorbents through an easy physical gelation of sodium alginate. The efficient biosorbents in the other study were prepared through an easy physical gelation of sodium alginate and carboxymethyl chitosan (CMCTS) in a decadic aqueous solution. The as-prepared hydrogel beads were not only able to demonstrate water superabsorbent properties and pH-responsible swelling properties but also showed good performance towards MB with maximum experimental adsorption capacity of 2518 and 1005 mg g^−1^, which is comparable with most reported lignocellulosic and alginate-based hydrogels.^62^

Tetrodotoxin or TTX can be adsorbed using peptidoglycan (PG) of lactic acid bacteria, but its complex structure and low solubility hinder its adsorption ability. In one particular study hydroxyl modifications of three PGs (A3a, A1g and A4a) were made through the selective oxidation of the primary hydroxyl group of the substrates by using 2,2,6,6-tetramethylpiperidine-1-oxyl or TEMPO. The carboxyl density of hydroxyl-modified PGs (HM-PGs) was observed to rise higher than 36 mmol g^−1^ and the surface electronegativity rose higher up to 59 mV when compared to those of the PGs. The adsorption capability of HM-PGs towards TTX was 1.48 µg mg^−1^, which was similar to the adsorption of the traditional adsorbent chitosan of aflatoxin B_1_ (1.39 µg mg^−1^). Besides, HMPGs reduced the toxicity of TTX from a highly toxic level to almost non-toxic, achieving a remarkable toxicity reduction of 99.85%.^71^ Following the treatment with HM-PGs, the neuronal cell model and the hippocampus of the mouse revealed that the residual TTX showed a lower level of neural injury and sodium channel blocking effect due to a lower neurotoxicity. The HM-PGs prepared in this experiment had a better adsorption capacity of TTX than emerging adsorbents like cyclodextrins and nerve cell membrane nano sponges, which formed the theoretical foundation of the design of effective and safe biological toxin adsorbents. The main shortcoming of biopolymers such as chitosan is that they can only be dissolved under acidic conditions, making them insoluble in alkaline conditions whereas the second weakness is that the surface area is low, limiting their ability to adsorb. Although the shortcomings of chitosan such as solubility at acidic and alkaline pH besides low surface area are notable, other biopolymers have the same problems. Native forms of cellulose and lignin usually are not very effective because of the rigid crystalline structure of the active sites, which are unreachable without considerable chemical treatment. Hydrogel-formers such as gelatin and alginate are plagued with excessive swelling and lack of mechanical stability which renders them hard to handle and regenerate. More specific polysaccharides such as β-d-glucan and peptidoglycan are restricted by their complicated and expensive isolation methods, which restrict their ability on an industrial or practical scale. Additionally, there exists a significant gap in research regarding their effectiveness against marine biotoxins, especially due to difficulties arising from elevated salinity levels and the varied spectrum of marine toxins. As a result, this field continues to be insufficiently explored and poorly recorded in the current literature.

Biopolymers are widely valued for being affordable and environmentally friendly, yet their unmodified forms come with notable drawbacks. Chitosan, for example, shows strong dependence on pH for its solubility and gel formation, while alginate-based hydrogels typically lack the durability required for repeated industrial applications. Overall, this category of materials often suffers from low surface area and variable performance, making them less effective than advanced, engineered adsorbents. The most critical gap in current research is the limited understanding of how these biopolymers behave against marine toxins in saline conditions. High concentrations of competing ions in seawater are expected to interfere with their adsorption efficiency, but this issue has not been sufficiently investigated. Therefore, future studies should prioritize affordable chemical modification strategies that improve stability, surface area, and resilience in complex environments, while still preserving the natural biodegradability of these materials.

### 2.2. Biohybrid and bioinspired adsorbents

Biohybrid materials based on biopolymers and nanostructures are used as adsorbents to increase the adsorption capacity and to overcome shortcomings such as difficult liquid recovery of a powdered adsorbent. One such example of biohybrid adsorbent is aerogel composite sponge of Lanthanum-based metal–organic framework embedded in cellulose aerogel or La-MOF@CA. These composite beads showed a huge high surface area of 1244.68 m^2^ g^−1^ that generates a high adsorption capacity of 710.75 mg g^−1^ to remove tartrazine dye.^63^ Solution casting methodology was used to prepare starch-HNT composite flakes which were then used in the adsorption of MB in aqueous solutions. XRD, SEM, FT-IR and TGA analyzed the sample flakes, and the surface area and point of zero charge (PZC) were determined with the help of the BET and PZC testing, respectively. The composites exhibited better adsorption of MB than the current starch-based composites with a maximum adsorption capacity of 604 mg g^−1^ in optimal conditions. Besides, the physisorption phenomenon was determined by the isotherm and kinetics models, higher temperature supported adsorption.^64^ Building on this concept of combining biopolymers with other materials, hydrothermal synthesis was used to formulate a novel biomaterial. To eliminate the anionic dye acid red 88 from synthetic wastewater, researchers synthesized an ecofriendly biosorbent CS-GLU/BCL using bacillus subtilis biomass and crosslinked chitosan-glutaraldehyde *via* hydrothermal and crosslinking methods.^54^ The BBD model based on a desirability tool established that the best conditions in the event of the extraction of AR88 (96.28%) by CS-GLU/BCL involve 0.7 g L^−1^ dose of adsorbent, 4.2 pH, and 29.6 min contact time.^44^

The synthesis and analysis of a novel biohybrid adsorbent, zinc and chromium layered double hydroxides or ZnCr-LDH/*Spirogyra* algae (ZnCr-LDH/Spi) was carried out and tested for the selective removal of the multi-dye mixtures of anionic dyes. Selectivity assays revealed that ZnCr-LDH/Spi specifically adsorb Direct Yellow (DY) as compared to other dyes. The adsorption kinetics followed pseudo-second-order model. The isotherm modeling corresponded with the Freundlich model which suggests multilayer adsorption on a heterogeneous surface. ZnCr-LDH/Spi demonstrated a maximum DY adsorption capacity of 48.780 mg g^−1^ at 313 K. The composite was found to regenerate up to five cycles with a percent removal of 56.37%, making it a promising option for sustained wastewater treatment applications.^65^

The B. subtilis HA-Ag_2_O nano-adsorbents have demonstrated significant potential for multi-functional applications in both photocatalytic environmental remediation and biomedical fields. Optimization of the key parameters such AgNO_3_ concentration and the reaction time led to the stabilization and enhanced effectiveness of these nano-adsorbents. Their potential as cost-effective and environmentally-friendly solution for wastewater treatment is particularly notable in terms of high photocatalytic efficiency with a dye degradation rate of 82.28% within 120 minutes.^66^ However, a major limitation of biohybrid adsorbents is the insufficient long-term stability under practical conditions. While current research often highlights high initial efficiency, it seldom examines material performance beyond a few regeneration cycles. Critical issues such as nanoparticle leaching, degradation of biopolymers by microbes in non-sterile environments, and the environmental impact from synthesis to disposal are largely unexplored. Without this essential data, the scale-up and commercial viability of these materials remains uncertain.

### 2.3. Agricultural and food wastes

Another example of a sustainable category of biobased adsorbents is biochar that is manufactured using agricultural waste. Nanoparticles can be integrated onto biochar, which is carbon rich and highly porous material synthesized by pyrolyzing organic waste. Biochar may also be used to disperse and stabilize nanoparticles to attain high adsorption capacity. One study demonstrated that spent coffee ground biochar pyrolysis at low temperature has eliminated 92% Remazol Brilliant Blue R dye.^67^ Following the theme of using food industry byproducts, grape pomace from the wine industry has also been investigated as a low-cost adsorbent. Grape pomace (approximately 15 million tons in the world) is one of the millions of tons of residues generated in the wine industry. Several processes have been proposed to be used in its application, but not many of them have concentrated on the adsorption of the mycotoxins. The pomace (pulp and skins) that is derived out of Primitivo grape has been proved to be a great aflatoxin adsorbent *in vitro*. The adsorption experiments were conducted at 37 °C and 1 µg of AFB_1_/mL was used in research. Overall, the authors discovered that big particles produced very low adsorption uptakes, but the adsorption slightly enhanced as the particle size was reduced. Moreover, the maximum adsorption was reached within 15 minutes. The efficiency of banana peels toward the *in vitro* removal of aflatoxins (AFB_1_, AFB_2_, AFG_1_ and AFG_2_) in 0.5 µg mL^−1^ of each toxin was investigated. Generally, dry banana peel dried in an oven was observed to be better in eliminating aflatoxins. Optimal adsorbent dosage was 60 mg mL^−1^ and the stationary phase was attained in 10 to 30 min. The banana peel extract efficiency rose with rise in pH (3–9). At pH 8, the highest adsorption uptakes of AFB_1_, AFB_2_, AFG_1_, and AFG_2_ were 74.9, 63.1, 76.1 and 92.8 respectively.^68^ While unprocessed agro-wastes like banana peels are useful, other studies have focused on more established materials like clays and processed biochars. Generally recommended as safe (GRAS) clays and biochar were tested for binding aflatoxin B_1_, ochratoxin A and zearalenone. The effect of surface area on the binding of the mycotoxin was examined. Surface area was observed to have a substantial effect on properties of binding. The *in vitro* tests demonstrated that pine biochar absorbed high concentration of mycotoxins in gastric fluid and the outcomes were similar to food grade activated coconut charcoal that is promoted as a detoxification supplement.^69^

The immobilization of lactobacillus plantarum (LP) in the interstitial spaces of the holocellulose, followed by a coating with the natural polymers (chitosan, Ch; and alginate, Al) resulted in the formation of Holo-LP/Ch/Al complex. Physicochemical examination confirmed that this complex was effectively immobilized and maintained stability at high bacterial concentrations. The ability of the complex to adsorb ochratoxin A (OTA) in wine model solutions was evaluated in terms of Box-Behnken design at different pH, time and concentration of the solutions. The findings revealed that optimal OTA removal, reaching a level of 53.68%, was attained at a pH of 3.0, with a contact duration of 75.39 minutes and a concentration of 43.82 mg mL^−1^.^68^ Moreover, biochar exhibited remarkable adsorption properties for removing microcystin-LR (MCLR), a common cyanotoxin generated by cyanobacteria and saxitoxin (STX), a potent marine neurotoxin. Under viable operating conditions such as dosage amount, contact time, initial concentration and initial pH, high percentage removal was observed. Moreover, biochar exhibited positive concomitant adsorption of both toxins, therefore, biochar can be a viable solution to water contaminated with both harmful algal bloom toxins. The adsorption data for both toxins were well-described by the Langmuir–Freundlich model, giving adsorption capacities of 3507.46 µg g^−1^ and 622.23 µg g^−1^ for MCLR and STX respectively. These findings imply that adsorption processes can comprise pore filling, hydrogen bonding, π–π interaction, hydrophobic, electrostatic as well as dispersive interactions.^70^ Samples of biochar were created at different pyrolysis temperatures of 400, 600, and 800 °C for a period of 60 minutes. With the rise in pyrolysis temperature, there was a notable increase in surface porosity, as specific surface area measurements increased from 7.26 ± 0.20 m^2^ g^−1^ to 408.2 ± 6.2 m^2^ g^−1^. Simultaneously, there was a significant decline in the levels of oxygen-containing functional groups, falling from 1518 ± 15 µg to 823.0 ± 7.7 µg. This research sought to examine how different adsorption factors, such as the biochar loading dosage, contact duration, initial concentration, and initial pH, influence the adsorption effectiveness of saxitoxin. These investigations demonstrated more than 90% toxin elimination at 0.01 g L^−1^ dosage rate, 30 min contact time and rising percent removal as initial STX levels and pH in water increased.^71^

Palm petioles were used to prepare biochar diphenylphosphine (DPP) biochar which was utilized to adsorb cationic Crystal Violet (CV) dye. DPP-biochar is a porous structure that was characterized by high carbon content and a well-developed porosity BET surface area of 640 m^2^ g^−1^. Adsorption process was highly influenced by solution pH (2.0–12) while independent on ionic strength. An adsorption equilibrium was reached within 15 min of contact. In summary, CV dye adsorption process on DPP-biochar was a complex set of numerous contributors. Major contributions include π–π interactions, pore filling, and hydrogen bonding and minor ones are electrostatic attraction and van der Waals forces.^72^ To form biochar-algal microspheres (BAM), sodium alginate was applied on biochar and scenedesmus obliquus, a type of microalgae that act as a biosorbent. Since microalgae are often difficult to recover from water as they are sensitive to environmental changes like pH, temperature, they were combined with biochar to improve recycling efficiency and stability. The adsorption capabilities of these materials towards malachite green (MG) were studied. The findings revealed 98% removal of MG with adsorption capacity of 1.98 mg g^−1^ at 25 °C.^73^

A biochar modified with NaOH, named NaCBC300, was created from corncobs *via* pyrolysis at 300 °C and later characterized to explore its adsorption characteristics and mechanisms for methylene blue (MB). The findings showed that NaCBC300 demonstrated remarkable MB adsorption performance, reaching a removal efficiency and adsorption capacity of 99.98% and 290.71 mg g^−1^, respectively, which are three to four times greater than those of unmodified biochar. This improved performance results from the higher concentration of hydroxyl groups and the creation of irregular flakes from the NaOH modification. The Freundlich isotherm analysis indicated that the adsorption process involving NaCBC300 and MB takes place in several layers.^74^

To produce an easy, low-cost and efficient biochar absorbent, activated biochar (KOH/PF-WB or biochar derived from PF-WB biomass and chemically activated with KOH) was prepared using phenol-formaldehyde (PF) resin modified wood utilizing a pyrolysis procedure followed by the activation with potassium hydroxide. According to the combined effect of PF resin and wood, KOH/PF-WB-7002-2 derived an outstanding porous form and rich oxygen-containing effective group, and accordingly, it had a high specific BET surface area (SBET) of 2301.61 m^2^ g^−1^, and overall pore volume (*V*_total_) of 1.205 cm^3^ g^−1^. Moreover, with a PF resin modification, more disorder and defect sites of activated wood biochar were obtained. Consequently, KOH/PF-WB7002-2 had a better adsorption capacity for Congo red (3472.22 mg g^−1^) and methylene blue (1112.35 mg g^−1^) dyes than other reported adsorbents. The pseudo-second-order kinetic model and Langmuir isotherm model provided a better fit for the adsorption process.^75^

A critical challenge in adsorbents based on agricultural and food wastes is the lack of standardized production procedures to guarantee consistency and safety of batches. Raw waste materials are not predictable in terms of their performance due to the inherent variability which makes them unreliable when it comes to industrial use where accurate results of treatment are required. The existence of specific byproducts, like Polycyclic Aromatic Hydrocarbons (PAHs), in biochar creation presents considerable health hazards because of their ability to leach. This issue is frequently disregarded, even though these materials, generally seen as sustainable, cannot be safely incorporated into food and water purification systems. To reduce these risks, it is crucial to develop standardized procedures that guarantee uniform quality and the elimination of toxic leachates.

## 3. Nanostructured adsorbents

### 3.1. Carbon based nanostructures

The nanomaterials composed of carbon exhibit outstanding electrical, chemical, and mechanical properties. Various carbon based materials such as carbon nanotubes (CNTs), activated carbon, graphene and its derivatives have been effectively utilized for the removal of water pollutants.^76^ This efficacy is largely due to their ultra-high surface area and microporous or mesoporous structures, which stem from the distinctive structural properties of carbon atoms. Graphene nanosheets including graphene, graphene oxides (GOs), and reduced graphene oxide (RGO), have been recognized as exceptionally efficient materials for adsorbing organic contaminants.^33^ This effectiveness is due to their polarized electron-rich and electron-poor regions, along with surface imperfections that promote the delocalization of π-electrons.^77,78^ These features greatly improve their adsorption ability, rendering them excellent options for environmental cleanup initiatives. A significant benchmark in this category is pyrrolidone-reduced graphene oxide which has been reported to achieve an exceptional adsorption capacity of 1698 mg g^−1^ for copper ions, highlighting the effectiveness of functionalized rGO in addressing multi-contaminant remediation challenges.^52^ Magnetic graphene oxides (MGO) and magnetic graphene are co-precipitated on GO/rGO nanosheets, enabling the identification and elimination of AFB_1_. The MGO adsorbent exhibited remarkable efficiency, effectively removing 88.82% of AFB_1_ from contaminated oil, showcasing its considerable potential for practical applications.^79^ Multi-class mycotoxins adsorption in solution by polyethyleneimine modified magnetic carbon nanotubes was reported to be an effective support for the analysis of multi-class mycotoxins in milk *via* LC-MS.^79^ Zhang *et al.* modified nano montmorillonite by introducing stearyl trimethyl ammonium bromide (STAB), leading to the formation of NMMT-STAB. This modified form demonstrated notably improved adsorption abilities compared to traditional NMMT when evaluated in dairy cow rumen fluid, attaining a 1.36-fold rise in decreasing AFB_1_ concentrations.^80^ The application of these magnetic carbon materials extends beyond mycotoxins. For instance, magnetic carbon core–shell nanoparticles are useful in getting rid of marine and freshwater toxins. The ability of adsorbents to adsorb is closely associated with their chemical and structural characteristics. For example, carbon-coated magnetic nanostructures can successfully remove nearly 70% of marine toxins without sulfate groups and up to 84.7% of cyclic peptide toxins present in freshwater solutions. This ability to adsorb is facilitated by the natural surroundings. Moreover, a sustainable technology called magnetic separation, especially employing multicore magnetite-based nanostructures, exhibits effective functioning and facilitates the total retrieval of the adsorbent materials.^19^ Carbon-based adsorbents are suggested for STX removal, as existing alternatives are not as efficient. Among these, mesoporous graphene nanoplatelets (GnPs) are notable as exceptional adsorbents, surpassing granular activated carbon (GAC) and other traditional materials in terms of adsorption kinetics and capacity. In particular, GnPs demonstrate an adsorption ability that is 93.5 times higher and a kinetic efficiency that is more than six times quicker compared to GAC.^81^

**Fig. 4:**
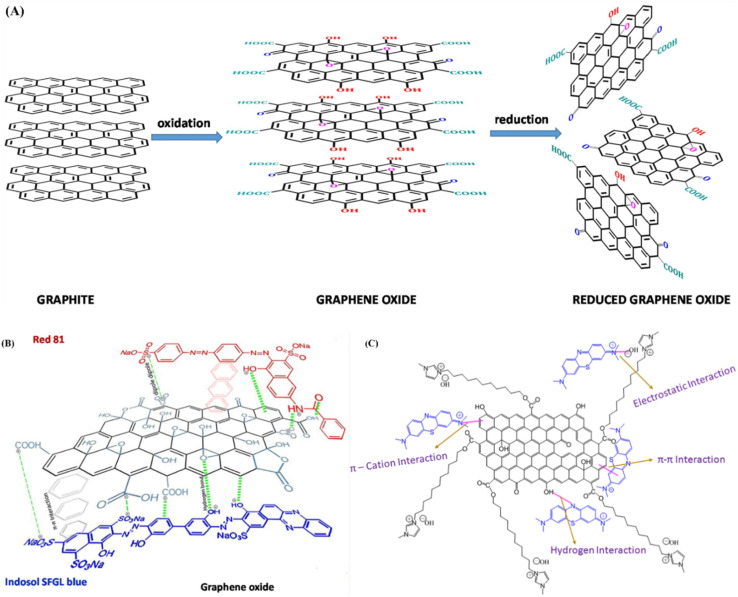
(A) Structures of graphite, graphene oxide and reduced graphene oxide (B) various chemical interactions involved in the adsorption of dyes by graphene. Reproduced with permission from ref. 46 (C) schematic illustration of the interaction pathways between GO-ImOH and MB, highlighting the roles of electrostatic attraction, π–π interaction between the graphene oxide framework and aromatic dye molecules, hydrogen bonding, and surface complexation *via* imidazole functional groups, collectively contributing to the enhanced adsorption performance. Reproduced with permission from ref. 82.

The exceptional adsorption capacity of graphene-based materials stems from a combination of intermolecular forces. These include π–π interactions, hydrogen bonding, and electrostatic interactions between dye molecules and the graphene oxide surface. However, the direct application of graphene oxide (GO) nanosheets encounters multiple issues, including restricted recyclability, inadequate separation, and the risk of undesirable residues in food items, making their practical use more complex. To tackle these challenges, the use of magnetic graphene as adsorbents by bonding Fe_3_O_4_ nanoparticles to GO or reduced graphene oxide (rGO) nanosheets enhances the effective separation and recovery of AFB_1_ from rice bran oils that are contaminated. Furthermore, advanced carbon materials such as carbon quantum dots, CNTs, and graphene oxide are gaining recognition as effective substitutes for conventional activated carbon in metal removal (see Fig. 4A). Among these, graphene oxide and its derivatives, such as reduced graphene oxide, functionalized graphene oxide, and graphene oxide composites have shown superior adsorption of uranium due to their unique 2D network structure and abundant surface functional groups. Graphene-based photocatalyst composites exhibit enhanced photocatalytic activity due to efficient transport of photogenerated electrons at the graphene–metal oxide heterojunction. This reduces electron–hole recombination and increases the production of reactive oxygen species, promoting the degradation and mineralization of organic pollutants. The differences in chemical interactions responsible for dye removal by commercial graphene and graphene oxide are illustrated in Fig. 4B.^39^

Fig. 4C shows that the binding of MB to graphene oxide modified with an imidazolium-based ionic liquid containing hydroxide groups (GO-ImOH) is characterized by different interactions. This conclusion is supported by results from FTIR, Raman spectroscopy, and microstructural investigations. Negatively charged hydroxyl, carboxylic, and phenolic groups in GO-ImOH enable strong electrostatic interactions with the cationic center of MB, which is the primary adsorption pathway. Additionally, π–π and cation–π interactions occur between the sp^2^ carbon domains of GO-ImOH and the aromatic rings or cationic sites of MB. Hydrogen bonding between oxygen-rich functionalities in GO-ImOH and nitrogen atoms in MB further contributes to adsorption. These chemical and structural features make GO-ImOH a highly efficient material for removing cationic dyes.^50^

A porous carbon-based magnesium aluminum pentafluoride 1.5-hydrate (MgAlF_5_·1.5H_2_O) nanocomposite was prepared from carbon-coated palygorskite *via* single-step HF etching. The material exhibited ultrahigh Congo red adsorption (4261 mg g^−1^), far surpassing porous carbon and uncoated palygorskite. Enhanced adsorption occurred between MgAlF_5_·1.5H_2_O and the SO_3_^−^ groups of the dye. The adsorbent proved to be low-cost, pH-independent, and highly efficient, making it ideal for wastewater dye removal, including emergency treatment scenarios.^50^ Literature survey reveals that although carbon-based nanostructures demonstrate remarkable performance, they encounter considerable practical and ecological challenges. A major issue is their elevated production expenses, combined with unsustainable synthesis techniques, like the modified Hummers' method for graphene oxide, which employs hazardous chemicals and demands substantial energy. Moreover, substances such as graphene and carbon nanotubes tend to clump together, leading to inadequate dispersion in water-based solutions unless extensive functionalization is applied. A significant concern is the challenge of isolating these nanoscale powders from treated media after adsorption, which often requires sophisticated and expensive filtration methods. Moreover, unresolved questions concerning their lasting environmental effects and possible nanotoxicity present regulatory and safety issues, obstructing their use in food and water systems.

Theoretical review of the literature has shown that there is a vast gap between the proven laboratory potential of carbon-based nanostructures and their preparedness in mass application. One of the biggest fields that are subject to further research is the establishment of scalable, cost-efficient and green pathways of synthesis. The current production system is not very industrially viable because of the high expenses and because of environmental issues. At the same time, there is a significant gap in knowledge about the long-term ecotoxicological effect and degradation routes of these nanomaterials in the actual environmental matrices. The areas that should be given priority in the future are to put the safety and economic systems that would help translate these high-performance materials into recommended and large-scale decontamination solutions.

### 3.2. Metal oxides

Metal oxides have attracted considerable interest because of their adaptability in numerous applications, such as wastewater treatment, photothermal methods, radiation therapy, tissue engineering, pharmaceuticals, antimicrobial properties, electronics, and optics.^83^ Investigators have concentrated on numerous essential traits of these substances, including their crystalline arrangement, dimensions, surface area, and form. Throughout the years, metal oxides and their composites have demonstrated significant potential as efficient adsorbents for the elimination of dyes from wastewater. Nanomaterials made from metal oxides demonstrate remarkable adsorption properties for various contaminants, owing to their plentiful surface-active sites, tunable surface chemistry, straightforward synthesis and functionalization, high surface area, affordability, and recyclability.^33^

Zinc nanoparticles act as efficient sterilizers due to their impressive chemical stability and outstanding photocatalytic characteristics, making them ideal companions with substances like copper oxide, titanium dioxide, cerium oxide, and iron oxide. ZnO is a noteworthy photocatalyst with a binding energy of 60 eV and a bandgap of 3.37 eV, thanks to its distinctive chemical and physical properties such as excellent electrochemical stability, robust oxidative ability, and minimal toxicity. Of the different metal oxides, ZnO is the most commonly used substance in heterogeneous photocatalysis. Interestingly, ZnO nanoparticles show a remarkable adsorption efficiency of 99.99% for Rhodamine B dye.^82^

Aluminum oxide NPs exhibit a corundum-like arrangement, in which one aluminum atom is encircled by six oxygen atoms. These nanoparticles display notable durability under extreme environmental conditions and retain outstanding chemical stability even at high temperatures, despite having low electrical conductivity. Their significant mechanical strength, large surface area, and affordability render them appealing for diverse applications, especially in biological environments because of their inert properties and the simplicity of surface modification. The performance of these oxides can be enhanced by attaching them to natural support materials. For example, applying synthesized Al_2_O_3_ nanoparticles to natural silica sand has shown remarkable stability and a considerable ability to eliminate methylene blue dye, facilitated by hydroxyl and amine groups on the nanoparticle surface that offer binding sites for effective dye adsorption.^84^ Among metal oxides, anodic aluminum oxide (AAO) is a notable variant that offers a viable choice for dye adsorption from water owing to its nanoporous configuration, extensive surface area, and chemical resilience. Produced *via* anodic oxidation, AAO presents considerable versatility for fabricating components with complex geometries, acting as fillers or essential elements in adsorption systems of different sizes. Studies on the removal of Eriochrome Black T (EBT) colored solutions have shown the efficiency of nanostructured AAO, illustrating its ability to adsorb EBT dye from water. The research indicated that the best dye removal took place after 2.25 hours at a stirring speed of 500 rpm and a temperature of 60 °C, reaching an impressive 99% removal effectiveness. Additionally, the adsorbent's reusability was verified, as it preserved more than 50% removal efficiency following four cycles.^85^

Bismuth, acknowledged as environmentally benign green metal and economically viable diamagnetic heavy metal, has been instrumental in the advancement of diverse NPs noted for their uniform structural, physicochemical, and compositional properties. Bismuth nanoparticles (BiNPs) display various beneficial characteristics, including strong X-ray attenuation, effective light-to-heat transformation, absorption in the near-infrared range, and efficient transmission over long distances. These qualities render BiNPs especially beneficial in uses such as integrated cancer treatment, photothermal and radiation therapies, multimodal imaging, drug transport, and biosensing. In-depth studies have been performed on bismuth oxyhalides (BiO_*x*_, with X indicating Cl, Br, or I) and bismuth chalcogenides, such as bismuth oxide, selenide, sulfide, and telluride, as possible therapeutic agents. Significantly, Bi_2_O_3_ has shown capacity to adsorb 63.9% of methylene blue. Moreover, Bi_2_O_3_ has inherent light-absorbing characteristics that allow it to fully break down dyes in sunlight, instead of just holding them on its surface. Future studies should focus on increasing its surface area and photocatalytic efficiency for better performance.^86^

Though bismuth NPs exhibit significant promise; however, titanium dioxide (TiO_2_) continues to be the most extensively studied metal oxide for decontamination applications. TiO_2_, a transition metal oxide, appears in four main varieties: anatase (tetragonal), brookite (orthorhombic), rutile (tetragonal), and monoclinic. Its appeal as a semiconductor photocatalyst arises from its remarkable physicochemical stability, minimal toxicity, cost-effectiveness, and efficiency in eliminating different pollutants from air and water. Like other semiconductors, TiO_2_ features a band gap, with anatase showing a 3.2 eV difference between the conduction and valence bands, whereas rutile has a band gap of 3.0 eV. Recent studies have investigated the integration of pure rutile TiO_2_ nanoparticles with microporous black clay (BC) to create a new nanocomposite designed for water purification through the adsorption of MB and methyl orange (MO) dyes. Analytical methods like X-ray diffraction, FTIR, and SEM were utilized to evaluate modifications in dye adsorption at different temperatures (250 °C, 400 °C, and 500 °C). The Rt/BC composite showed exceptional separation efficiencies of 96.7% for MO and 91.4% for MB at pH levels of 3.0 and 8.0, respectively, with a surface adsorbent density of 100 mg g^−1^. Significantly, MO molecules clustered more swiftly than MB, leading to greater adsorption capacities, suggesting distinct adsorption interaction for these two dyes.^33^ The test was conducted in purified water where only one dye either MO or MB was dissolved in water. The wastewater generated by industrial activities is not just a combination of salts; it consists of a complicated mixture of dyes and organic substances. This complexity implies that different components may compete for adsorption sites, which could significantly decrease the effectiveness of titania nanoparticles in pollutant removal. The research has determined ideal pH levels for TiO_2_ effectiveness; pH 3.0 for methyl orange and pH 8.0 for methylene blue, but the response of these particles at a neutral pH of 7.0, typical in natural water sources and various industrial discharges is still unclear. The mechanism of adsorption is mainly due to electrostatic interactions; however, additional factors like pore filling, hydrogen bonding, and π–π interactions could also be involved. In a similar investigation, cubic phase magnesium oxide nanoparticles were produced to efficiently remove harmful dyes such as Congo red (CR) and toluidine blue (TB), attaining more than 98% removal efficiency at a concentration of 25 ppm in an acidic setting (pH 3–4) using 0.2 g of MgO nanoparticles. The maximum load capacities for CR and TB were found to be 136 mg g^−1^ and 132 mg g^−1^, respectively. Isotherm analysis showed that the Freundlich model more accurately reflects the experimental equilibrium data, indicating a heterogeneous surface of the nanoparticles, while kinetic studies demonstrated that the adsorption process adheres to a pseudo-second-order rate.^87^ Based on the metal oxides nanoparticles, due to four unpaired electrons in the 3d shell, iron oxides or magnetic alloys of iron oxides are of interest to many researchers due to their highly distinctive properties which include superparamagnetic and high magnetic moments.

Nanoparticles of iron oxide have become of great significance because they are magnetically recoverable and can easily form composite with silica and carbon to increase adsorption capability. Two types of magnetic nanostructured particles, which are synthesized using wet chemistry techniques, were prepared in a study with the use of magnetite nanoparticles, m-Fe_3_O_4_ NPs, and other inorganic parts (carbon or mesoporous silica) to promote chemical affinity between the toxins. m-Fe_3_O_4_ coated with carbon NPs, m-Fe_3_O_4_ @C and mesoporous silica matrices with Fe_3_O_4_ NPs overgrowing the surface were prepared as m-Fe_3_O_4_ and mesoporous-Si, through a one-step solvothermal process and a soft template method, respectively. To deliver a good magnetic response in terms of separation, both nanostructured materials were done using a magnetic multicore method. In both systems, the magnetic cores are coated, leaving the carbon or silica surfaces and internal pores available for toxin adsorption, maximizing binding sites. The adsorption properties of the composite of two varieties of magnetite-based nanostructures paired with different inorganic carbon forms and mesoporous silica demonstrated considerable efficacy in eliminating mycotoxins. The utilized nanoparticles are superparamagnetic, marked by low coercivity and remanence in their magnetization curve, improving their colloidal stability and enabling a strong magnetic response in moderate magnetic fields. This characteristic enables the quick removal of nanoparticles from the liquid medium with an external magnet, demonstrated by their fast attraction to the magnet in just seconds during experimental trials. To confirm this, the researchers initially evaluated the capacity of carbon and mesoporous silica particles to remove both hydrophilic and lipophilic toxins by treating polluted aqueous solutions with 250 mg of nanoparticles (125 mg L^−1^) for 60 minutes. Although silica particles were ineffective against hydrophilic toxins and azaspiracids, they successfully attained a 43% removal rate for yessotoxin. As a result, carbon nanoparticles were chosen for additional investigation into their adsorption capabilities across different types of toxins. Nonetheless, the efficacy of carbon-based nanoparticles might decrease in natural water bodies because of competing molecules such as humic and fulvic acids, which can disrupt the adsorption locations on the carbon composites.^1^

Notable adsorption of Congo red and malachite green was observed with tetragonal morphology copper oxide nanoparticles, employing monolayer adsorption and heterogeneous surface interactions. The adsorption characteristics adhered to both Langmuir and Freundlich isotherm models. Nonetheless, it is essential to acknowledge that this research was performed in regulated laboratory settings, which may not truly represent the intricacies of actual wastewater systems.^87^ In comparison, magnetic nanoparticles based on polyhedral oligomeric silsesquioxanes exhibited enhanced adsorption properties for aflatoxins, efficiently eliminating these pollutants from cereal samples.^87^ In this case the incorporation of polyhedral oligomeric silsesquioxane (POSS) greatly enhanced the surface density of functional groups on magnetic nanoparticles. This improvement resulted in a significant increase in the accessibility of active binding sites. As a result, magnetite (Fe_3_O_4_)nanoparticles coated with POSS and polyionic liquid–polystyrene (PIL–PSt), known as Fe_3_O_4_@POSS@PIL–PSt, can efficiently promote hydrophobic, π–π, and electrostatic mixed-mode interactions for the binding of aflatoxins in cereal samples.^17^ An efficient magnetic adsorbent (Fe_3_O_4_@ATP) was prepared using the precipitation method by dispersing Fe_3_O_4_ nanoparticles onto the natural attapulgite (ATP) and later using it as an adsorbent for aflatoxin B_1_ removal from contaminated oil. This magnetic composite had a high capacity to remove AFB_1_ from contaminated oil with 86.82% removal efficiency using 0.3% dosage. The paramagnetic properties of Fe_3_O_4_@ATP, showing a saturation magnetization of 50.86 emu g^−1^, allowed for convenient separation with an external magnet. The adsorption process effectively described by the Freundlich isotherm followed the pseudo-second-order model.^88^ Nonetheless, the effective use of metal oxide nanoparticles is limited by their physicochemical instability in intricate aqueous settings. A major constraint is their limited effective pH range, affected by surface charges, frequently restricting their performance to certain acidic or alkaline conditions that fail to represent neutral effluents seen in practical situations. This requires expensive pH modifications. Furthermore, the propensity of these nanoparticles to cluster in solutions greatly diminishes their active surface area, creating worries about the release of metal ions, which compromises their safety for the environment. Future studies must focus on creating surface-modified metal oxides that show improved stability and effectiveness over a wider pH range, as well as extensive investigations aimed at reducing metal ion leaching to guarantee their safe use.

### 3.3. Nanocomposite materials

Surface functionalization acts as an effective approach to improve the performance of nanomaterials in adsorption tasks. Silica nanoparticles modified with nitrophenyl furfural have shown an impressive capability to eliminate tartrazine dye, reaching an adsorption capacity of 203.5 mg g^−1^, owing to their significant surface area of 80 m^2^ g^−1^. The adsorption process features monolayer adsorption, represented by the Langmuir isotherm and adhering to pseudo second-order kinetics.^89^ Congo red is effectively adsorbed with the use of chitosan activated carbon. Optimal dye to adsorbent ratio was reported to be 1 : 10, hence 1 g adsorbent and 100 ppm dye at pH = 7 gave 100% dye removal from aqueous system at room temperature. Thermal activation refers to the process through raising of temperature and electrostatic interaction through acidic pH that can help a process be more efficient in removal. Since removal efficacy is high in acidic pH hence it restricts its use in neutral or basic pH. Surface functionalization can be utilized to improve the use of nanomaterials as adsorbents in the removal of a variety of contaminants, such as synthetic dyes, mycotoxins, and marine toxins.^87^ Beyond traditional single-mode removal, cutting edge progress in nanostructure design is increasingly moving towards multifunctional materials that integrate adsorption based capture with catalytic degradation.

A major advancement in this area is the fabrication of three component composites such as g-C_3_N_4_/MnO_2_/Pt, which effectively captures organic contaminants due to their highly porous structure.^98^ This composite material achieved eight times increase in degradation rate compared to the binary composites (g-C_3_N_4_/Pt) through enhanced separation of electron–hole pairs. Nonetheless, their dependence on Pt imposes significant economic limitations that impede their widespread industrial use. Moreover, a significant research gap persists regarding the stability of active sites in complex real-world settings; existing studies predominantly concentrate on idealized aqueous conditions, thus neglecting the performance of these ternary systems in the negative circumstances of high salinity and surface fouling, especially within food-related wastewater, which remains largely unexplored.^90^ In addition to it, bismuth oxyhalides represent a unique class of layered photocatalysts with conveniently tunable band gaps. Their distinct structure facilitates the generation of an internal electric field which significantly increases the separation efficiency of electron–hole pairs which are photogenerated enabling high activity in remediation of aquatic contaminants.^91^

**Fig. 5:**
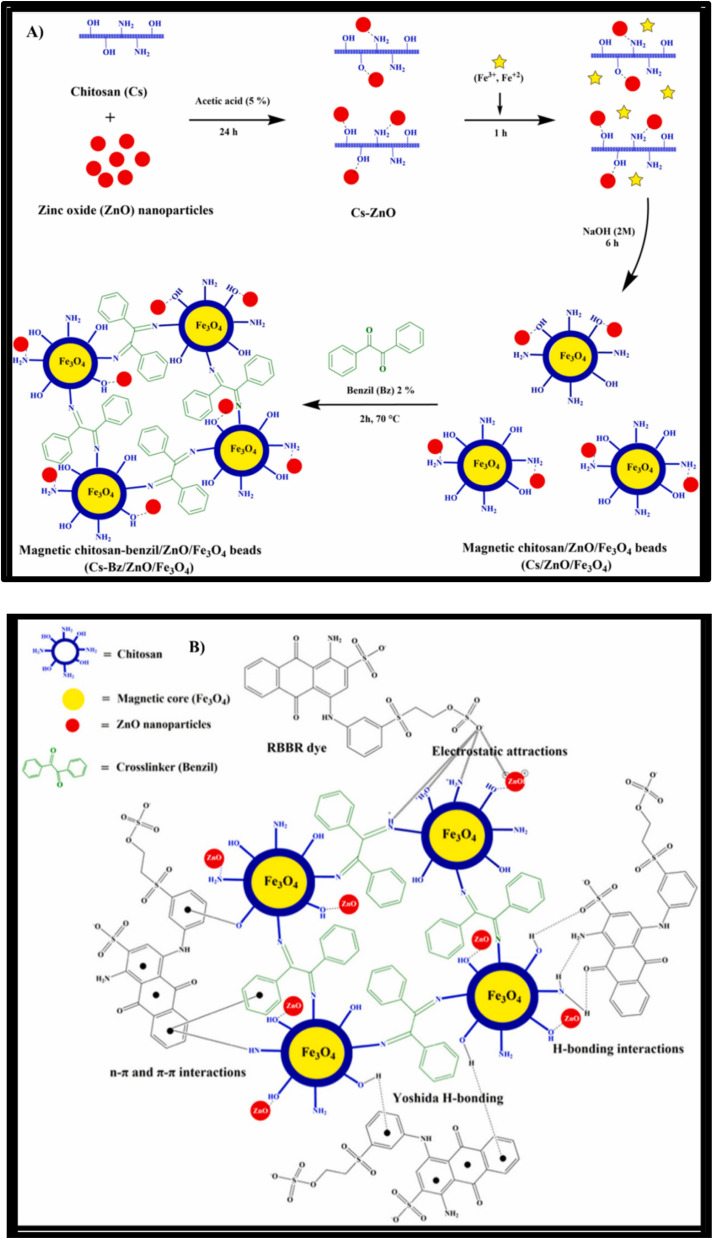
(A) Schematic illustration of the stepwise synthesis of the Cs-Bz/ZnO/Fe_3_O_4_ nanocomposite, highlighting the integration of chitosan-based functionalization with ZnO and magnetic Fe_3_O_4_ components (B) proposed adsorption mechanism of RBBR on the Cs-Bz/ZnO/Fe_3_O_4_ surface, illustrating the contributions of electrostatic interactions, hydrogen bonding, and π–π interactions between dye molecules and surface functional groups, along with the role of ZnO and Fe_3_O_4_ in enhancing adsorption efficiency and facilitating magnetic separation. Reproduced with permission from ref. 92.

Thermal activation pertains to the method of increasing temperature and utilizing electrostatic interactions at acidic pH to enhance the efficiency of a removal process. Due to high removal efficacy in acidic pH, its application is limited in neutral or basic pH. Leaching or aggregation of iron components off nanocomposite through the repeated cycles can be a limiting factor for its removal capacity. A prime example of a multifunctional magnetic nanocomposite is the chitosan-benzil/ZnO/Fe_3_O_4_ (Cs-Bz/ZnO/Fe_3_O_4_) system. The multi-step synthesis process for these beads is outlined in Fig. 5A. Furthermore, Fig. 5B details the proposed adsorption mechanism for RBBR dye onto the nanocomposite surface, highlighting the roles of electrostatic attraction and H-bonding. The Cs-Bz/ZnO/Fe_3_O_4_ nanocomposite contains multiple active adsorption sites that enable various interactions with Remazol Brilliant Blue R (RBBR) molecules. Electrostatic attraction is the dominant mechanism, arising from positively charged surface groups (–NH_3_^+^, –OH_2_^+^, C=HN^+^, and Zn(OH)^+^) interacting with the –SO_3_^−^ groups of RBBR. Hydrogen bonding also occurs between surface hydrogen atoms of the adsorbent and nitrogen or oxygen atoms in RBBR. Additionally, Yoshida hydrogen bonding, n–π, and π–π interactions contribute through interactions between aromatic rings of the adsorbent and dye molecules. These combined interactions result in a significantly enhanced adsorption capacity of 620.5 mg g^−1^ for RBBR.^58^

Currently, the use of magnetic physical adsorbents for detoxification is widely applied in the food industry, however, the fabrication of high-efficiency low-cost absorbents without damaging the nutritional quality of food is a major challenge. Herein, a simple, green, efficient, and cost-effective method for the magnetic solid-phase extraction of aflatoxin B_1_ from edible oils and aqueous matrices was developed using a dopamine-loaded biomass chitosan–iron–cobalt spinel oxide nanocomposite (DC/CFOS NC). The adsorption characteristics of DC/CFOS aligned with pseudo-second-order kinetics (*k*_2_ = 0.199 g mg^−1^ min^−1^) and Freundlich isotherm models (*K*_f_ = 1.139 (mg g^−1^) (L mg^−1^). Benefiting from its high specific surface area, microporous structure, and polar/non-polar active sites, the as-prepared DC/CFOS exhibited appealing adsorption performance for AFB_1_ (50.0 mg mL^−1^), as measured using the Freundlich isotherm model. The mechanistic studies demonstrated that the synergistic effects of the surface complexation and electrostatic interactions between the functional groups of DC/CFOS NC and AFB_1_ were the dominant adsorption pathways.^35^

A hierarchical fungal mycelia@graphene oxide@ Fe_3_O_4_ (FM@GO@ Fe_3_O_4_) was fabricated as an efficient adsorbent for simultaneous removal of aflatoxin B_1_(AFB_1_). FM@GO@ Fe_3_O_4_ could be reused more than 10 times with the high adsorption efficiency. Compared to non-magnetic adsorbents, FM@GO@ Fe_3_O_4_ could be easily separated by an external magnet, demonstrating the utility and practicality of the prepared hierarchical nanocomposites. The adsorption capacity of FM@GO@ Fe_3_O_4_ for AFB_1_ was 0.3533 µg mg^−1^. Hydrogen bonding, π–π interactions, electrostatic interaction and Hydrophobic interactions were the possible driving forces for the adsorption of AFB_1_.^93^

Besides graphene-based composites, metal–organic frameworks (MOFs) and their derivatives represent another group of highly porous substances being examined for environmental uses. Among these, Cu-BTC is notable as a prominent MOF, acclaimed for its considerable specific surface area and porosity, which makes it efficient in eliminating environmental contaminants. However, Cu-BTC framework could not remain stable under humid conditions because of the structure collapse caused by the replacement of the organic linker by water molecules. In contrast, Cu-BTC MOF-derived porous carbonaceous materials have better chemical stability. Furthermore, these materials retain the high porosity of the original MOF after the calcination and chemical etching, which could offer more adsorption sites. AFB_1_ adsorption capacity of three porous adsorbents increased rapidly within 180 min, and the AFB_1_ adsorption rate of C-Cu-BTC MOF 800 and C-Cu-BTC MOF-600 were found faster than C-Cu-BTC MOF-400 at the beginning, which indicated that C-Cu-BTC MOF-800 and C-Cu-BTC MOF-600 have more available unoccupied active adsorption sites for adsorption at the primary stage. Furthermore, for C-Cu-BTC MOF-800 and C-Cu-BTC MOF-600, the contact time required to reach adsorption equilibrium were 90 and 120 min, respectively, whereas, the adsorption of AFB_1_ on C-Cu-BTC MOF-400 was much slow and the adsorption equilibrium reached after 840 min.^94^

**Fig. 6:**
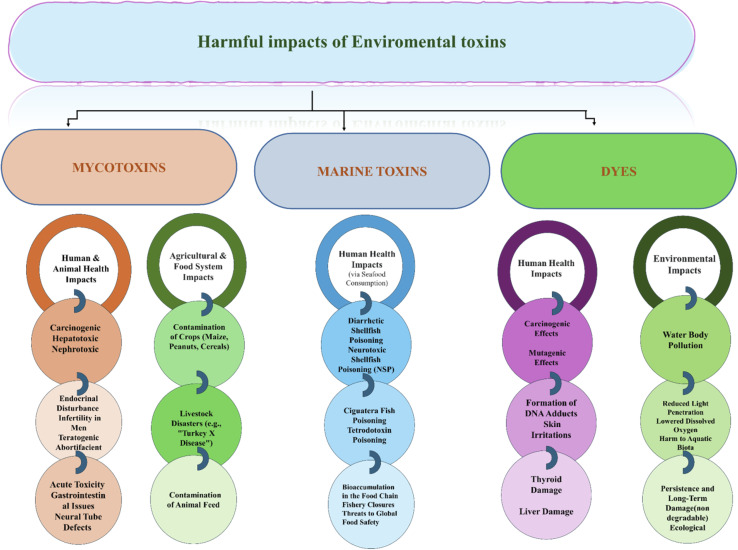
Schematic illustration depicting the sources and environmental distribution of mycotoxins, marine toxins, and synthetic dyes, along with their pathways of exposure and their combined pathological, agricultural, and ecological impacts on human health, food safety, and environmental systems.

Fig. 6 illustrates the detrimental effects of environmental toxins, with a particular emphasis on mycotoxins, marine toxins, and dyes. Mycotoxins mainly affect agriculture and food systems, causing crop contamination, livestock diseases, and serious human health effects such as carcinogenicity, endocrine disruption, and acute toxicity. Marine toxins pose major risks to human health through seafood consumption, leading to illnesses like shellfish poisoning, ciguatera, tetrodotoxin poisoning, and bioaccumulation in the food chain. Dyes primarily cause human health issues such as carcinogenic, mutagenic, and thyroid effects, while also polluting water bodies, reducing oxygen levels, harming aquatic life, and persisting long-term in the environment. Parallel to the terrestrial threats posed by mycotoxins, marine biotoxins represent a significant and growing concern for global food safety, particularly within the seafood industry. However, the application of adsorption-based remediation strategies for marine toxins presents a unique set of challenges not always encountered with dyes or mycotoxins. This difficulty stems primarily from the vast chemical diversity within the phycotoxin group, which includes both small, water-soluble hydrophilic compounds like saxitoxin and large, complex lipophilic molecules such as okadaic acid. Furthermore, the complex matrices in which these toxins are found, namely high-salinity seawater and biological tissues introduce competitive ions and organic matter that can significantly hinder adsorbent performance.

## 4. Adsorbent performance across different matrices

The true measure of an adsorbent's utility is found in how it operates in intricate, real-world settings. While foundational studies often use idealized aqueous solutions, the goal is decontamination of matrices like industrial wastewater or food products, each presenting unique challenges. This section evaluates the efficacy of bio-based and nanostructured adsorbents, first in aqueous systems and then considering the more complex challenges posed by food matrices.

### 4.1. Aqueous systems

The textile sector, which employs approximately 65 million individuals, is the second-largest employer globally after agriculture. It uses a variety of dyes that contribute to the growing issue of wastewater contamination. A case study by Mohamed *et al.*,^95^ highlights the successful application of a carbon nanotube/bentonite @ polyvinylidene fluoride tri-flouro ethylene (MWCNTs/BT/P(VDF-TrFE)) nanocomposite for the elimination of Congo red dye from wastewater. This study provides a model for understanding adsorbent performance in aqueous system. The nanocomposite exhibited high adsorption capacity, reaching a maximum monolayer adsorption capacity of 143.88 mg g^−1^. Furthermore, adsorption process was found to adhere to pseudo second order kinetics. In another study, iron oxide-flax seed-based hybrid nanocomposites were synthesized and were utilized and examined for the adsorption of malachite green. Experimental outcomes proved that the composite achieved high removal efficiency, as 90% of malachite green was obtained from a 10.0 mg L^−1^ solution, with 1.0 g L^−1^ of the composite, within 15 minutes at 30 °C and pH 7.^96^

A simple and cost-effective approach to enhance wastewater treatment has been developed using molybdenum-doped niobium pentoxide (Mo–Nb_2_O_5_). The analysis highlighted an increase in surface-active oxygen species and defect sites. Mo doping significantly improved adsorption capacities for cationic dyes, with MB and CV showing increases to 35.4 and 44.8 mg g^−1^, respectively, while adsorption for CR and TC decreased due to selective affinity for cationic pollutants. Kinetic studies confirmed chemisorption as the dominant mechanism, with the adsorption process favoring PSO kinetics.^97^

### 4.2. Food matrices

Applying adsorbents for decontamination in food matrices introduces a significantly higher level of complexity compared to aqueous systems. The goal is to remove harmful contaminants like mycotoxins or marine toxins without compromising the nutritional value or safety of the food product. The development of adsorbents specifically designed for this purpose is therefore a critical area of research. Food matrices are intricate mixtures of proteins, lipids, carbohydrates, and minerals, which can interfere with the adsorption process by competing for active sites or causing adsorbent fouling.

In one particular study, adsorbent was synthesized using a green method from renewable and waste-based raw materials, lignin from coconut coir, chitosan from shrimp shell waste, and iron nanoparticles synthesized using black tea leaf extract. The final nanocomposite of L-CS-Fe showed maximum adsorption of AFB_1_ up to 95.6% by utilizing a small amount of adsorbent (1 mg mL^−1^) with a wide range of structural stability. The synergistic effects enhanced by the composite formation of lignin, chitosan, and iron resulted in enhanced adsorption properties, leveraging the individual strengths of each component; lignin's adsorptive capacity, chitosan's biodegradability and chelation properties, and iron's reactive capabilities.^55^ Another study demonstrates, polydopamine modified nanofibers membrane (PDA-PS NFsM) which combines the advantages of nanofiber membrane and PDA, was used as an adsorbent for removal of five aflatoxins (AFB_1_, AFB_2_, AFG_1_, AFG_2_ and AFM_1_) from several representative liquid foods including edible oil for non-polar liquids, soy sauce and milk for polar liquids, rice vinegar for mildly acidic liquids, and liquor with different ethanol content, demonstrating excellent detoxification effect. Not only were the conditions of the whole adsorption process mild, but the nanofiber membrane can also be directly taken out by tweezers after the adsorption was completed. The removal efficiency for every single aflatoxin from all samples involved above was more than 76.5% within 1 h at 25 °C, except the liquors with higher ethanol content, for which the efficiency was lower.^98^

### 4.3. Practical applicability and scale-up considerations

Despite the significant progress in nanostructured and bio-based adsorbents development, their practical implementation for the purification of water and food matrices remains limited. A major challenge exists in the difference between adsorption efficiency seen in controlled lab conditions and that in actual systems. Most studies are conducted using single-component synthetic solutions, which do not adequately represent the complexity of actual wastewater or food matrices. In real environments, the presence of competing ions, natural organic matter, and matrix-specific components such as proteins, lipids, and carbohydrates can significantly hinder adsorption efficiency by occupying active sites or inducing fouling.^99,100^ Consequently, adsorption capacities reported under idealized conditions are often overestimated compared to practical scenarios.

Regeneration and reusability are critical parameters for evaluating the economic and environmental feasibility of adsorbents. While many studies report successful regeneration using approaches such as solvent washing, pH adjustment, or thermal treatment, these evaluations are typically limited to a small number of adsorption–desorption cycles. In many cases, a gradual decline in adsorption capacity is observed due to incomplete desorption, structural degradation, or loss of active sites.^101,102^ Therefore, long-term stability assessments over extended cycles are essential to validate the durability of these materials for continuous use. Table 1 shows practical applicability of various adsorbent materials, highlighting performance metrics such as the tested matrix, number of effective regeneration cycles, specific system type used, and notable operational or technical limitations hindering industrial implementation.

**Table 1:** Practical applicability of various adsorbent materials, highlighting performance metrics such as the tested matrix, number of effective regeneration cycles, specific system type used, and notable operational or technical limitations hindering industrial implementation

Adsorbent type	Matrix tested	Regeneration cycles	System type	Key limitation	Ref.
Activated carbon (bio-based)	Real wastewater	3–5 cycles	Batch	Reduced efficiency due to NOM	99
Chitosan-based adsorbents	Synthetic + real water	4–6 cycles	Batch	Structural instability after reuse	100
Agricultural waste-derived adsorbents	Wastewater	2–4 cycles	Batch	Limited durability	101
Graphene oxide composites	Synthetic solutions	3–5 cycles	Batch	High cost, scalability issues	105
Carbon nanotubes	Synthetic water	3–4 cycles	Batch	Aggregation, regeneration loss	102
Biochar-based adsorbents	Real wastewater	5–10 cycles	Batch/column (limited)	Fouling, variable performance	104
Fixed-bed systems (various adsorbents)	Wastewater	Continuous	Column	Breakthrough limitations	103
Nanocomposites (metal oxide-based)	Synthetic + limited real samples	3–6 cycles	Mostly batch	Scale-up challenges	106

A key factor to consider is the shift from batch adsorption experiments to continuous-flow systems. Batch studies, while useful for initial assessment, do not truly represent industrial treatment methods. Continuous systems, such as fixed-bed columns, provide more realistic insights into adsorbent performance under dynamic conditions, including flow rate, residence time, and breakthrough behavior.^103^ However, investigations employing continuous-flow configurations remain scarce for many emerging adsorbents, particularly bio-based and nanocomposite materials.^104^ Expanding research in this area is mandatory for bridging the gap between laboratory findings and large-scale applications.

Scalability and cost-effectiveness further influence the practical deployment of adsorbents. Bio-based materials derived from agricultural and food wastes offer distinct advantages due to their low cost, abundance, and sustainability.^100^ In contrast, the synthesis of nanostructured adsorbents often involves complex procedures, expensive precursors, and energy-intensive conditions, which may limit their large-scale production.^105^ Overall, future research should prioritize the evaluation of adsorbents under realistic conditions, including multi-component systems and complex matrices, while also addressing regeneration efficiency, continuous operation, and cost considerations. Such efforts are essential to advance the transition of high-performance adsorbents from laboratory-scale studies to practical applications.

## 5. Comparative analysis and emerging trends

The pursuit of effective adsorbents for removing dyes, mycotoxins, and marine toxins has evolved significantly over the decades. While modern research focuses on novel materials, it is crucial to analyze them in the context of traditional adsorbents that have long served as the industry baseline. This section provides a comparative analysis, contrasting the performance of traditional materials with modern bio-based and nanostructured adsorbents, and identifies the key emerging trends driven by the need to overcome long-standing limitations. The selection of an adsorbent is a complex decision balanced between efficiency, cost, selectivity, and environmental impact. By comparing different classes, a clearer picture of their respective roles emerges. For many years, materials like activated carbon (AC), bentonite clay, and zeolites have been the workhorses of adsorption technology. Their primary strengths are their low cost, widespread availability, and proven effectiveness for general-purpose purification. Activated carbon is renowned for its vast microporous structure and extremely high surface area, making it a highly effective adsorbent for a broad spectrum of organic pollutants, including many dyes.

Despite their utility, traditional adsorbents suffer from several critical limitations that hinder their use in sensitive or advanced applications. Poor selectivity is arguably their biggest drawback, especially for food applications. Activated carbon is a non-selective adsorbent; it binds indiscriminately to a wide range of molecules. When used to remove mycotoxins from food, it also adsorbs essential nutrients like vitamins, minerals, and amino acids, thereby reducing the nutritional value of the product. Powdered forms of these adsorbents (*e.g.*, powdered activated carbon, bentonite clay) have excellent kinetics due to their high surface area, but they are notoriously difficult to remove from the liquid phase after treatment. This necessitates additional, often costly, post-treatment steps like centrifugation or fine filtration, complicating the overall process. While effective, they often require long contact times to reach equilibrium, making them less suitable for rapid, continuous-flow industrial processes. The regeneration of saturated activated carbon typically requires high-temperature thermal processes, which are energy-intensive and can damage the adsorbent's structure, leading to a loss of efficiency over cycles. Chemical regeneration can be effective but often produces hazardous secondary waste streams. Bio-based adsorbents (a sustainable alternative) represent a direct response to the sustainability concerns of traditional adsorbents. Materials like chitosan, lignin, and biochar from agricultural waste are abundant, renewable, low-cost, and biodegradable and offer good removal efficiencies.^31,107^ particularly for dyes, by leveraging their natural porous structures and functional groups. But in their raw form, their adsorption capacities are often lower than that of high-grade activated carbon or nanostructured materials. Their performance can be inconsistent, and they may have poor mechanical strength, making them less robust for repeated use in industrial settings compared to materials like zeolites. Tables 2–4 summarize the adsorption performance of various adsorbents for the removal of dyes, marine and aqueous toxins respectively, from different matrices, including removal efficiency, isotherm and kinetic models, optimal operating conditions, maximum adsorption capacity, and contact time.

**Table 2:** Summary of adsorption performance of various adsorbents in aqueous matrix for the removal of dyes from different matrices, including removal efficiency, isotherm and kinetic models, optimal operating conditions, maximum adsorption capacity (*q*_max_) and contact time^a^

Adsorbent	Adsorbate	Optimal condition for adsorbate				*q*_max_ (mg g^−1^)	Contact time (min)	Removal efficiency (%)	Isotherms/kinetics	Model fitting method	*R* ^2^ (isotherm model)	Ref.
		Dose G	*C*_0_ (mg L^−1^)	pH	Temp. K							
Ziziphus spina-christi seeds AC	Methylene blue	0.6	500	—	—	666.66	30	98.62	Langmuir/pseudo-second order	Linear	0.986	42
Moringa stenopetale seed husk AC	Methylene blue	0.055	316	—	—	436.68	19.3	99.4			0.954	
Burmese grape seeds AC	Methylene blue	—	60	—	—	163.9	40	Not specified			0.983	
Commercial granular activated carbon (GAC)	Tartrazine	2	5	2	296	10.20	60	99.81	Langmuir/Pseudo-second-order	Linear	0.993	43
Rice husk (RH)		4	5	2	296	6.87	120	90.45			0.983	
Framework@calcium alginate aerogel composite sponge	Tartrazine	0.02	—	4	328	710.75	100	Up to 98.7	Langmuir/pseudo-second-order	Non-linear	—	63
Spent coffee ground biochar (SCGB)	Remazol Brilliant Blue R (RBBR)	0.20	74.7	2	—	Not specified	120	95.4	Not specified	Not specified	Not specified	67
NiFe-layered double hydroxide supported metal–organic framework-embedded alginate bead composite	Tartrazine	1.33 mg mL^−1^	8.02	3.74	∼298	Not specified	283	94.82	Langmuir–Freundlich/pseudo-second-order	Non-linear	—	110
Orange peel powder (sulfuric acid activated)	Tartrazine	0.2	40	3	298	122.25	∼20	Not specified	Temkin/pseudo-second-order	Linear	0.999	113
Orange peel powder (thermally activated)						121.74					0.999	
Orange peel powder (soda activated)						116.35					0.999	
Surfactant-modified zeolite (SMZ-CPC)		0.08	30	10	298	5.06	30	> 94	Sips/Elovich	Non-linear	—	118
Magnetite@PEI (Fe_3_O_4_@PEI)	Ponceau 4R	0.0026	—	5–10	298	204	5	95–99	Langmuir/pseudo-second-order	Linear	0.992	119
Magnetite@chitosan (Fe_3_O_4_@CS)	Azorubine	0.0029		5–8	298	142	10	95–99			0.996	
Polyamide (PA) nanofibers	Sunset yellow	∼20	—	3	298	39	30	∼95			0.999	
Magnetic chitosan composite (m-CS-c-PAM)	Sunset yellow	1	20	—	298	359.71	60	Not specified	Langmuir/pseudo-second order	Linear	Not specified	120
Chitosan-vermiculite beads	Brilliant blue	5 g L^−1^	500	10.9	298	181.6	1440					
Brewers' spent grains-activated carbon	Tartrazine	2	10	3	308	32.15	60	100	Langmuir/Elovich	Linear	Not specified	
Gelatin/montmorillonite nanocomposite	Malachite green	—	350	9	—	950.5	45	Not specified	Langmuir isotherm	Non-linear	—	121
Graphene/ZnO/hydroxyapatite nanocomposite	Azo dyes	—	—	—	—	700	4	94.8–96.5	Langmuir/chemisorption	Linear	0.982	122
Carbonized bamboo leaves powder (citric acid treated)	Methylene blue	0.2	400	7.5	305	725	60	Not specified	Langmuir	Linear	Not specified	123
Spent tea leaves		0.7	65	9	303	300.05	120		Not specified	Not specified	Not specified	
Michelia figo leaves (MFL)		—	200	11	298	238.10	180		Langmuir/pseudo second order	Linear	Not specified	
Cassava sievate biomass AC	Tartrazine	0.1	150	1–2	303–313	20.83	90	Not specified	Freundlich/pseudo-second-order	Linear	0.999	124
	Sunset yellow					0.091					0.926	
Zinc–aluminum layered double hydroxide (Zn–Al-LDH)	Tartrazine	0.4	40	5.8	298	282.48	60	96.83	Langmuir/pseudo-second-order	Linear	0.990	125
Polyamidoamine (PAMAM) dendrimer gel		0.02	1500	7	293	689.7	60	Not specified	Langmuir/pseudo-second-order	Linear	>0.980	
AF-CMS (amine-functionalized cellulose microGraphenes)		—	500	4	318	618.65	30				>0.990	
Layered double hydroxide/Diatomite (LDH/DIA)		0.01	20	8	293	555.6	60				0.990	
Magnesium aluminum azelate layered double hydroxide	Tartrazine	0.1	—		∼298	173.2	1440 (24 h)	99.9	Langmuir	Linear	0.999	126
β-cyclodextrin/chitosan/PVA composite hydrogel film	Tartrazine	0.005	—	20 mg L^−1^	∼298	1436.04	180	96.3	Langmuir/pseudo-second-order	Non-linear	—	127
β-Cyclodextrin/chitosan/PVA composite hydrogel film	Congo red	0.01		7	∼298	366.34	120	95.38			—	
Activated carbon from bovine rumen waste	Tartrazine	0.025	—	3	318.15	111.90	60	99.96	Freundlich/pseudo-first-order	Non-linear	—	128
Silica xerogel modified with cetyltrimethylammonium bromide (SiO_2_-CTAB)	Tartrazine	0.020	—	6.8	297	9.62	60	Not specified	Langmuir/pseudo-second-order	Linear	0.740	129
Dialdehyde-functionalized nanocellulose fibrils/chitosan/aminated metal–organic framework aerogel (NDC)	Tartrazine	0.5 g L^−1^	100	3	303	689.66	720	98.56	Langmuir/Pseudo-second-order	Non-linear	—	130
EGDE-crosslinked chitosan film (0.32 mm thickness)	Tartrazine	0.024–0.030	—	2.5	303	873.37	1500	Not specified	Sips/pseudo-first-order	Non-linear	—	131
Manganese–iron layered double hydroxide modified polyvinylidene fluoride (PVDF) polymer membrane	Tartrazine	0.015	16	5	∼298	20.97	15	94.74	Langmuir/pseudo-first-order	Linear	0.963	132
Manganese–iron layered double hydroxide modified polyviylidene fluoride (PVDF) polymer membrane	Erythrosine B	0.015	13	13 mgL^−1^	∼298	6.15	15	92.13			0.996	
Magnetic graphene oxide-semicarbazide (MGO-Se)	Tartrazine	2	50	3.5	298	33.2	9	91.7	Langmuir/pseudo-second-order	Linear	0.998	133
Magnetic graphene oxide-semicarbazide (MGO-Se)	Sunset yellow	2	50	3.5	298	29.8	9	92.8			0.991	
Sweet potato residue-derived activated carbon (SPAC)	Carmine	0.1	5–70	7	323	468.1	180	Not specified	Sips/pseudo-second-order	Non-linear	—	134
	Tartrazine		5–250		323	267.4					—	
	Allura red AC		5–70		323	252.6					—	
Treated avocado seed (TAS)	Basic yellow 28	0.075	—	10	293–323	49.30	60	89.93	Temkin/pseudo-second-order	Non-linear	—	135
Iron doped activated carbon from *Delonix regia* bark	Methylene blue	1	50–250	6	298	357.14	90	>99	Langmuir/pseudo-second-order	Linear	0.998	136
Iron doped activated carbon from *Delonix regia* bark	Tartrazine	1	50–250	3	298	147.06	60	>98			0.999	
Cola nut shell AC (phosphoric acid activation) (potassium hydroxide activation)	Tartrazine	0.5	5–30	2	∼298	24.57	5	Not specified	Langmuir/pseudo-second-order	Linear	0.981	137
		0.5	5–30	2	∼298	21.60	10	Not specified			0.982	
Doum palm seed shell activated carbon	Rhodamine B	1	—	10	303	88.40	40	Not specified	Freundlich/pseudo-second-order	Linear	0.99	138
	Methylene blue		—	12	333	81.81	30				0.98	
	Methyl orange		—	2	333	73.38	90				0.97	
Cobalt-impregnated clinoptilolite (15% co)	Lanaset green B		—	∼7	298	152.51	60		Langmuir/pseudo-second-order	Non-linear	Not specified (standard error) *σ* = 0.001–0.019	
Fly ash/polyacrylic acid/melamine (FA/PAA/M) composite	Sudan II		1–30	2–8	296	142.3	∼70	95 (1st cycle)	Langmuir/pseudo-second-order	Non-linear	—	139
ZnO–Al_2_O_3_-rGO/MWCNT (photocatalyst/Adsorbent)	Sudan III	0.6	—	5.5	298	220	∼90	>98% (calculated from *q*_max_)	Langmuir/pseudo-second-order	Non-linear	—	140
Nelumbinis stamen activated carbon (NSAC)	Sudan red G	0.4	5–15	3	323	36.19	16	92.13	Langmuir/pseudo-second-order	Linear	0.988	141
Nelumbinis stamen activated carbon (NSAC)	Sudan I					35.64		95.57			0.974	
Nelumbinis stamen activated carbon (NSAC)	Sudan II					34.60		90.06			0.961	
Nelumbinis stamen activated carbon (NSAC)	Sudan red 7B					38.97		91.75			0.994	
Antimony-based ionic liquid (M–Sb)	Sudan II	0.75	0.75	—	—	162.1	40	>90	Langmuir/pseudo-second-order	Non-linear	—	142
Bismuth-based ionic liquid (M–Bi)				—	—	170.0					—	
Pristine biochar from rice husk	Eriochrome Black T (EBT)	1.5	—	2		Not specified	120	94	Not specified	Not specified	Not specified	143
Natural diatomite (ND)	Carmoisine	1	50	2	290	12	30	Not specified	Freundlich/pseudo-second-order	Linear	∼0.99	144
Graphene oxide-chitosan-Fe (GO-CS-Fe) nanocomposite	Sudan I	1	350	4	310	360.6	90	99.1	Langmuir/pseudo-second-order	Non-linear	—	145
	Sudan II					353.7		98.3			—	
	Sudan III					351.0		97.7			—	
Nanosized scoria	Carmoisine	200	45	7	318	54.42	17 280 (12 days)	>95	Not specified	Not specified	Not specified	146
Activated carbon from raphanus seeds solid residual (ACRS)	Methylene blue (MB)	1	10	7	318	8.6	120	∼99	Langmuir/pseudo-second-order	Linear	0.968	147
Graphene oxide-carboxymethyl cellulose-*co*-acrylamide (GO/P(CMC-Co-Am)) nanocomposite	Azure C (basic violet 3)	1	50	5	290	9.14	120	Not specified	No isotherm model applied/pseudo-second-order	Not specified	Not specified	148
Polyaniline/Bi_2_O_3_ (PANI/Bi_2_O_3_) nanocomposite	Alizarin red	1	10	11.4	303	9.006	100	95	Langmuir/pseudo-second-order	Non-linear	—	149
Forsterite nanoparticles	Evans blue	1	10–99	3		42.3	10	∼100	Langmuir/pseudo second-order	Linear	0.996	150
Chrysotile	Methylene blue (MB)	0.14	419	13	298	352.97	5.5	∼99	Langmuir/pseudo-first-order	Non-linear	—	151
Lizardite		0.1	449			254.85	15		Freundlich/pseudo-first-order		—	
Activated carbon from guava seeds	Tartrazine	12	20	6.10	298	1.62	80	97.6	Elovich/pseudo-second-order	Linear	0.983	152
Activated sawdust from pinus halepensis wood		0.05	—	2		127.74	60	99.52	Langmuir/pseudo-second-order	Non-linear	—	153

a All adsorption capacities are reported in mg g^−1^, and concentrations are expressed in mg L^−1^. Where necessary, units have been converted for consistency. ‘—’ indicates data not reported in the original study or *R*^2^ values omitted for non-linear entries to ensure comparability. Parameter estimation in the majority of cited literature is based on linear transformations; however, modern standards advocate for nonlinear fitting due to its higher reliability. As a comprehensive review, our primary goal is to compile and present the existing body of data as originally reported in the literature. To maintain data integrity while avoiding the mathematical inconsistency of comparing coefficients with different *Y*-axes, *R*^2^ values are listed only for linear isotherm studies. All cross-methodological evaluations of adsorbent performance are strictly based on absolute adsorption capacities(*q*_max_). Values represent reported single values; standard deviations (±) are omitted to maintain table uniformity across the vast range of cited literature where such data was largely unavailable.

**Table 3:** Summary of adsorption performance of various adsorbents for the removal of Marine toxins from different matrices, including removal efficiency, isotherm and kinetic models, optimal operating conditions, maximum adsorption capacity (*q*_max_), contact time, and reusability^a^

Adsorbent	Adsorbate	Matrix	Removal efficiency%	Isotherms/kinetics	Fitting model method	*R* ^2^	Reusability	Optimal conditions				*q*_max_ (mg g^−1^)	Contact time (min)	Ref.
								Dose G	*C*_0_ (mg L^−1)^	pH	Temp. K			
Graphene nanoplatelets (GnPs)	Saxitoxin (STX)	Aqueous solution/simulated field water	>90	Langmuir/pseudo-second-order (physisorption chemisorption)	Non-linear	—	—	0.0020	—	7	298	0.05124	60	81
Hydroxyl-modified peptidoglycan (HM-A4α)	Tetrodotoxin (TTX)	Fish muscle extract	99.8	Not specified	Not specified	Not specified	—	0.003	5	—	310	0.00148	60	154
Polyaluminium chloride-modified clay	Brevetoxin 2	Aqueous solution	98	Freundlich/pseudo-second-order	Linear	0.87	Good (reusable for 3 cycles with some loss in efficiency)	0.10	0.01	7.24	293	6.41	120	155
Carboxyl-functionalized covalent organic polymer (TpPa-COOH)	Saxitoxin (STX)	Natural freshwater	∼99.7	Langmuir/pseudo-second-order (physisorption & chemisorption)	Linear	0.99	Good (reusable for at least 3 cycles)	0.00011	—	6–7	292	5.69	60	156

a All adsorption capacities are reported in mg g^−1^, and concentrations are expressed in mg L^−1^. Where necessary, units have been converted for consistency. ‘—’ indicates data not reported in the original study or *R*^2^ values omitted for non-linear entries to ensure comparability. Parameter estimation in the majority of cited literature is based on linear transformations; however, modern standards advocate for nonlinear fitting due to its higher reliability. As a comprehensive review, our primary goal is to compile and present the existing body of data as originally reported in the literature. To maintain data integrity while avoiding the mathematical inconsistency of comparing coefficients with different *Y*-axes, *R*^2^ values are listed only for linear isotherm studies. All cross-methodological evaluations of adsorbent performance are strictly based on absolute adsorption capacities (*q*_max_). Values represent reported means; standard deviations (±) are omitted to maintain table uniformity across the vast range of cited literature where such data was largely unavailable.

**Table 4:** Summary of adsorption performance of various adsorbents for the removal of mycotoxins from different matrices, including removal efficiency, isotherm and kinetic models, maximum adsorption capacity (*q*_max_), contact time, and reusability^a^

Adsorbate	Matrix	Removal efficiency (%)	Isotherms/kinetics	Model fitting method	*R* ^2^ (isotherm model)	Reusability	*q*_max_ (mg g^−1^)	Contact time (min)	Ref.
Bentonite/humic acid/beta-glucan-mannan composite (70 : 10 : 20)	Simulated gastrointestinal fluid	98.07%	Pseudo-second-order (chemisorption)	Linear	Not specified	Not specified	0.332	360	45
LS-Fe_3_O_4_@UiO-66	Aqueous solution of AFB1	∼90%	Freundlich/pseudo-second-order	Linear	0.976	Excellent; >80% efficiency after 3 cycles	Not specified for the composite (focus was on overall removal)	180	47
MGO (magnetic graphene oxide)	Contaminated oils (rice, bran oil)	98.41%	Freundlich/pseudo-second-order	Linear	0.9910	Excellent	1.68	60	79
Stearyl trimethyl ammonium bromide modified nano-montmorillonite (NMMT-STAB)	Dairy cow rumen fluid	Not specified	Freundlich/pseudo-second-order	Linear	0.95	Not specified	9.23	15	80
C-Cu-BTC MOF-600	Vegetable oils (peanut, corn)	>90%	Langmuir/pseudo-second-order	Non-linear	—	Not specified	16.92	30	94
OP-bentonite (orange peel extract modified bentonite)	Buffered solutions and simulated gastrointestinal fluids	∼95%	Langmuir/pseudo-second-order	Linear	0.992	Not specified	166.00	120	107
Metformin-chitosan/silica-cobalt ferrite nanospheres (Mt-CS/CFS NSs)	Aqueous solution & cow's milk	>91%	Freundlich/pseudo-second-order	Linear	0.993	Excellent (∼88% efficiency after 7 cycles)	3.89	80	112
Fe/N-PC (derived from Fe-doped ZIF-8)	Aqueous solution of AFB_1_ (also tested in tofu wastewater)	99.88%	Langmuir/pseudo-second-order (adsorption); PMS-activated catalytic oxidation	Linear	0.999	Good; >75% efficiency after 3 cycles	202.80	30	157
Walnut shell nano-biosorbent (WSN)	Aqueous solution of AFB_1_	93.7%	Langmuir/pseudo-second-order	Linear	0.9950	Excellent; <5% drop in removal after 5 cycles	176.30	45	158
Sodium alginate incorporated attapulgite (SA-ATP)	Aromatic peanut oil	98.37%	Langmuir/pseudo-second-order	Linear	0.9925	Not specified	0.0406	60	159
Sugarcane bagasse fly ash (BFA)	Simulated gastrointestinal fluid (buffer solutions)	∼91%	Langmuir/pseudo-second-order	Non-linear	—	Not specified	115.00	120	160
Chitosan/palygorskite (C-A1)	Simulated gastrointestinal fluid (PBS buffer)	∼95.5%	Linear isotherm model/pseudo-second-order	Linear	0.982	Stable with minimal desorption	1.89	20	161
HKR-90 (hexadecyltrimethylammonium bromide modified kaolinite)	Phosphate buffer solution conditions	∼97%	Langmuir/Freundlich/sips	Non-linear	—	Not specified	8.90	30	162

a All adsorption capacities are reported in mg g^−1^, and concentrations are expressed in mg L^−1^. Where necessary, units have been converted for consistency. ‘—’ indicates data not reported in the original study or *R*^2^ values omitted for non-linear entries to ensure comparability. Parameter estimation in the majority of cited literature is based on linear transformations; however, modern standards advocate for nonlinear fitting due to its higher reliability. As a comprehensive review, our primary goal is to compile and present the existing body of data as originally reported in the literature. To maintain data integrity while avoiding the mathematical inconsistency of comparing coefficients with different *Y*-axes, *R*^2^ values are listed only for linear isotherm studies. All cross-methodological evaluations of adsorbent performance are strictly based on absolute adsorption capacities. Values represent reported means; standard deviations (±) are omitted to maintain table uniformity across the vast range of cited literature where such data was largely unavailable.

**Fig. 7:**
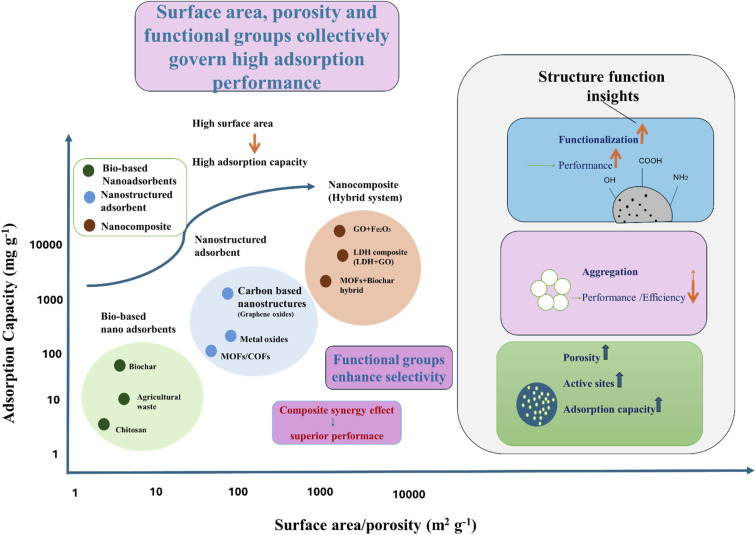
Conceptual representation of the relationship between structural properties (*e.g.*, surface area, porosity, and functionalization) and adsorption performance of bio-based, nanostructured and nanocomposite adsorbents, depicting how material design affects adsorption capacity and efficiency toward emerging contaminants.

Nanostructured adsorbents (The high-performance Frontier) like graphene oxide, MOFs, and metal oxide nanoparticles are designed to overcome the performance ceilings of traditional adsorbents. Their key advantages are exceptionally high surface area, rapid kinetics, and tunable surface chemistry, which allows for enhanced selectivity. For example, the precise pore engineering of MOFs can create binding sites tailored to specific mycotoxin molecules, a level of selectivity unattainable with activated carbon. The integration of magnetic nanoparticles (Fe_3_O_4_) directly addresses the critical separation problem of powdered adsorbents, allowing for near-instantaneous recovery with a simple magnet. Their main hurdles are high manufacturing costs, complex synthesis procedures, and potential nanotoxicity. The environmental fate of nanoparticles that may leach from the adsorbent is a significant concern that requires thorough investigation before they can be safely deployed in food and water systems. The most promising direction, evident throughout this review, is the creation of hybrid materials. These composites strategically address the limitations of all other classes. For instance, coating low-cost biochar with magnetic nanoparticles combines sustainability and easy separation. Functionalizing a traditional clay with specific organic molecules can enhance its selectivity for target toxins. This approach leverages the strengths of each component to create a final product that is more effective and practical than the sum of its parts. Fig. 7 demonstrates the relationship between structural characteristics and adsorption efficiency, revealing that increased surface area, porosity, and functionalization significantly enhance adsorption capacity, particularly in nanocomposite systems due to synergistic effects. Current trends also indicate a significant global shift towards green nanotechnology, with over 142 000 research articles by 2022 highlighting the transition to bio-nanohybrids.^108^ Among these, cellulose nanocrystals derived from biomass have demonstrated their ability to address critical challenges in the industry, such as the degradation caused by chlorine in desalination membranes.^52,108^

While numerous studies report high adsorption capacities and favourable kinetics, a critical evaluation reveals several limitations in the interpretation of adsorption models. Many investigations depend on linear shape of isotherm models, such as Langmuir and Freundlich, which can introduce significant error due to distortion of the error structure and unequal weighting of data points. As a result, nonlinear fitting techniques are more frequently recommended because they provide more precise parameter estimates and more accurately represent the true adsorption behaviour. Moreover, kinetic analyses frequently rely on simplified pseudo-first-order or pseudo-second-order models without proper validation against diffusion-controlled processes, potentially leading to a misinterpretation of the rate-limiting steps involved.^103^ A key obstacle encountered during this comparative review was the lack of raw experimental data from the referenced literature, which hindered a comprehensive non-linear recalibration of all parameters. To mitigate this issue, we adopted a classification based on a ‘fitting method.’ It is important to note that *R*^2^ values derived from linear transformations cannot be directly compared to those from non-linear fits due to variations in *Y*-axis parameters. Consequently, to ensure data integrity and avoid invalid mathematical comparisons, we have reported *R*^2^ values solely for linear studies as provided by the original authors, while our analysis primarily emphasizes absolute adsorption capacities as the universal metric for evaluating adsorbent performance across different statistical frameworks. By integrating the diverse data presented in Tables 2–4, this study identifies the ternary hybridization framework as the most effective approach for multi-matrix decontamination. As proposed by Koffi Sossou and coworkers, this framework provides a robust solution for field applications by synergistically combining a biological foundation for sustainability, nanostructures for high selectivity and magnetic components for efficient recovery, thereby ensuring high performance even in complex, real-world aquatic system.^109^ This comparative analysis shows that bio-based adsorbents ensure sustainability,^60^ nanostructures provide high selectivity^110^ and integration of magnetic components^22^ enable efficient recovery. This three-part synergy enables the development of stable and matrix-tolerant materials that perform effectively in complex systems. The move towards industrial sustainability is embodied in the “waste-to-resource” method. Industrial aluminum swarf can be transformed into effective adsorbents for wastewater treatments and reused for soil stabilization later, supporting a circular lifecycle.^111^ Additionally, advanced sophisticated characterization techniques such as XPS and VSM remain important for evaluating selectivity of adsorption and magnetic recovery of these materials.^36^ However, translating this ternary framework into large-scale application requires addressing reported matrix-level obstacles. A deeper evaluation states that while high removal efficiencies are common in ideal de-ionized water based systems, removal efficiencies often decline in real-world saline systems^19^ due to high ionic strength and surface fouling by proteins and lipids derived from food.^35^ To address this issue, researchers are developing advanced adsorbents capable of simultaneously removing metals and emerging contaminants such as PFAS. However, Sossou *et al.* highlight that the effectiveness of these materials is significantly influenced by factors like pH and the complexity of the surrounding matrix.^109^ Bridging this gap requires a transition from batch experiments to continuous-flow pilot studies, ensuring that these bio-nanohybrids maintain their performance under sustained hydraulic conditions.^108^ These uncertainties are aggravated by a lack of long-term reliable data on nanoparticles leaching and environmental fate.^23^ Identifying and resolving these limitations is the next crucial step for establishing truly standardized and matrix-resilient decontamination techniques.

The practical significance of hybrid materials relies on their longevity and capability for industrial expansion. Along with having high capacities, these materials need to show the capability to maintain performance through multiple regeneration cycles to be suitable for real world applications.^61,112^ However, a significant research gap still exists in transitioning from laboratory-scale batch experiments to the continuous flow systematic treatments standard in large-scale purification. For bridging this gap, structural durability of bio-nanohybrids under constant hydraulic load is required while simultaneously minimizing production costs, by using low-cost agro-waste precursors.^67,113^ Incorporation of life cycle assessments into materials design can lead future research to cost effective and scalable decontamination solutions.

## 6. Safety, environmental implications and regulatory considerations

The successful application of nanostructured adsorbents in water purification and food systems relies on a thorough assessment of their safety, environmental effects, and the changing regulatory landscape. The toxicological properties of these substances are affected by numerous physicochemical factors, including chemical makeup, surface area, particle morphology, and stability in suspension. As particle size decreases, the surface-to-volume ratio significantly increases, thereby boosting their reactivity and potentially resulting in an overproduction of reactive oxygen species (ROS). The increased generation of ROS can lead to oxidative stress, causing cellular damage when nanomaterials are consumed from contaminated sources.^114^ Dietary exposure to nanoparticles happens not only due to deliberate application but also through environmental discharge and incorporation into the food chain. When consumed, these substances may interact with the intestinal epithelium and possibly compromise barrier integrity. Existing safety frameworks suggested by the European Food Safety Authority (EFSA) highlight a hypothesis-based and tiered method for risk assessment.^115^

This procedure starts with evaluating the breakdown and solubility of substances under simulated gastrointestinal circumstances to assess their durability in nanoparticulate form. If the materials show acceptable durability, additional *in vitro* studies are conducted to explore possible cytotoxic effects. Since intestinal absorption is affected by particle size, with uptake happening for particles up to around 250 nm, it's crucial to create nanostructured adsorbents that are either biologically stable or completely recoverable. This method seeks to reduce the likelihood of systemic distribution.^116^ The inadvertent release of nanoscale adsorbents during purification process can lead to secondary pollution. Unlike other materials, nanomaterials are highly mobile and highly persistent in the environment, enabling their transfer across air, water and soil systems leading to accumulation within food chain, particularly in aquatic life. Therefore, extensive ecotoxicological monitoring is important to evaluate their long-term impacts as they move from environmental sources into food commodities.^114^

Currently there is a lack of harmonized global regulations specifically governing the use of nanotechnology in food and water decontamination. While the FDA and EFSA offer general guidance, specific standards for “nanomaterials contact” materials are still evolving. To attain these objectives of UN Agenda 2030 for sustainable development, recent studies highlight a “safer-by-design” approach which focuses on reducing hazards during material synthesis, disposal and use. To evaluate impacts from production to end-of-life, future research should prioritize comprehensive life cycle assessment. Integrating chemical analysis along with ecotoxicological testing is important to develop threat indicators, ensuring public trust and regulatory acceptance by policymakers and environmental managers.^115,117^

## 7. Conclusion and future outlook

The effective removal of contaminants from water and food is a critical challenge, and adsorption has proven to be an exceptionally versatile and powerful tool. This review demonstrates that both bio-based and nanostructured adsorbents have significant promise for the elimination of dyes, mycotoxins, and marine toxins from aqueous systems and food matrices. Bio-based materials have benefits in sustainability, cost-effectiveness, and environmental compatibility, whereas nanostructured adsorbents deliver increased surface area, adjustable functionality, and improved adsorption efficiency. The efficacy of these materials is significantly affected by adsorption mechanisms, material structure, and matrix intricacy, underscoring the necessity for system-specific design. A comparative examination indicates a growing interest in hybrid and nanocomposite materials that integrate sustainability with elevated adsorption capacity. Subsequent research must prioritize scalability, regeneration, selectivity within intricate matrices, and practical application to enhance technology for the decontamination of safe water and food.

For decades, traditional adsorbents like activated carbon, clays, and zeolites have been the cornerstone of purification technologies. Their low cost and general effectiveness made them indispensable. However, their significant drawbacks, most notably their lack of selectivity, the difficulty of separating fine powders from treated media, and the high energy cost of regeneration have created a clear and persistent need for superior alternatives. Activated carbon-based systems remain the leading option for adsorbents in commercial applications. In parallel, novel materials such as nanocomposite membranes and functionalized biochar are gradually moving towards pilot-scale and industrial applications. The research detailed in this review represents a direct and successful response to these limitations. Bio-based adsorbents have emerged as a sustainable, low-cost alternative, transforming agricultural waste into valuable resources. More powerfully, nanostructured adsorbents have shattered the performance ceilings of their traditional predecessors, offering unprecedented adsorption capacities, rapid removal rates, and critically, the potential for tailored selectivity. The development of magnetic nanocomposites stands out as a particularly elegant solution, directly solving the long-standing issue of adsorbent recovery. Modern advancements in the field are defined by the emergence of hybrid systems, which synergistically combine sustainability, high performance, and practical applicability in a way that no single class of material could achieve on its own. Regulatory approval for adsorbent materials requires compliance with safety and leaching standards established by agencies such as the EPA, EFSA, and WHO.

While scientific progress is undeniable, the path forward requires a concerted effort to address the practical hurdles that separate laboratory success from large-scale industrial and environmental application. It is imperative to move beyond testing in clean, single-contaminated water. Future studies must rigorously evaluate adsorbent performance and stability in the “messy” reality of industrial wastewater, food matrices (like milk, oils, and juices), and natural water bodies. This is the only way to prove their robustness against fouling and competitive adsorption. The “wonder materials” of today will remain a laboratory curiosity unless they can be produced affordably on a large scale. Research must focus on simplifying synthesis processes, reducing energy consumption, and utilizing low-cost or waste-derived precursors for both bio-based and nanostructured materials.

The use of nanomaterials in food and water carries a profound responsibility. Rigorous, long-term studies on nanotoxicity, biocompatibility, and the potential for leaching are not optional, they are essential for regulatory approval and public trust. Comprehensive Life-Cycle Assessments (LCAs) are needed to ensure that new adsorbent technologies are truly “green” from cradle to grave. The next frontier, especially for food applications, is the development of adsorbents that can act with surgical precision. The goal is to design materials that can, for example, selectively bind aflatoxin molecules while leaving all vitamins and minerals untouched. This will require advanced techniques like molecular imprinting and bio-functionalization. Future work must look beyond 3–5 regeneration cycles and investigate the long-term durability of adsorbents. The focus should be on developing regeneration techniques that are low-cost, low-energy, and do not produce harmful byproducts, thus closing the loop and creating a truly sustainable decontamination cycle.

## Conflicts of interest

Authors declare no conflicts of interest.

## Abbreviations

AC: Activated carbon
AMX: Amoxicillin
AOPs: Advanced oxidation processes
APTES: 3-Aminopropyl triethoxysilane
BPB: Bromophenol blue
CB: Conduction band
CDs: Carbon dots
CIP: Ciprofloxacin
CMC: Carboxymethyl cellulose
CNTs: Carbon nanotubes
CTAB: Cetyltrimethylammonium bromide
CV: Crystal violet
CVD: Chemical vapor deposition
DCs: Diclofenac sodium
EBT: Eriochrome Black T
EC: Emerging contaminants
FAAS: Flame atomic absorption spectrometry
FT-IR: Fourier transform infrared spectroscopy
GEM: Gemfibrozil
GO: Graphene oxide
GQDs: Graphene quantum dots
HNTs: Halloysite nanotubes
HPLC-MS: High-performance liquid chromatography-mass spectrometry
HWW: Hospital wastewater
IBP: Ibuprofen
ICP-MS: Inductively coupled plasma-mass spectrometry
LCA: Life cycle assessment
LDH: Layered double hydroxide
LOD: Limit of detection
MB: Methylene blue
MFS: Magnetic flax seeds
MG: Malachite green
MNMs: Magnetic nanohybrid materials
MNPs: Magnetic nanoparticles
MNZ: Metronidazole
MO: Methyl orange
MOFs: Metal–organic frameworks
NPs: Nanoparticles
OG: Orange G (dye)
PAHs: Polycyclic aromatic hydrocarbons
PARA: Paracetamol
PCBs: Polychlorinated biphenyls
PDMS: Polydimethylsiloxane
PEG: Polyethylene glycol
PFC: Perfluorinated compounds
PFOA: Perfluorooctanoic acid
PFOS: Perfluorooctanesulfonic acid
POM: Polyoxometalate
PPCPs: Pharmaceuticals and personal care products
PTSA: *p*-Toluenesulfonic acid
PVDF: Polyvinylidene fluoride
rGO: Reduced graphene oxide
RhB: Rhodamine B
SEM: Scanning electron microscopy
SPIONs: Superparamagnetic iron oxide nanoparticles
SWCNTs: Single-walled carbon nanotubes
TAM: Tamoxifen
TC: Tetracycline
TEM: Transmission electron microscopy
TEOS: Tetraethyl orthosilicate
TGA: Thermal gravimetric analysis
TOC: Total organic carbon
UV: Ultraviolet
VB: Valence band
VSM: Vibrating sample magnetometer
WCA: Water contact angle
XPS: X-ray photoelectron spectroscopy
XRD: X-ray diffraction

## Data Availability

No primary research results, software or code have been included and no new data were generated or analysed as part of this review.
